# Computational Studies toward the Identification of CB2R-M1R Dual Modulators

**DOI:** 10.1021/acsomega.5c07866

**Published:** 2026-02-19

**Authors:** Israa H. Isawi, Rufaida Al-Zoubi, Rima Hajjo, Rayan M. Obeidat, Islam H. AlKhawaldeh, Omar M. Al Kilani, Mahmoud J. Alhaj Hasan, Paula Morales

**Affiliations:** † Department of Medicinal Chemistry and Pharmacognosy, Faculty of Pharmacy, 108609 Jordan University of Science and Technology, Irbid 22110, Jordan; ‡ Department of Pharmacy, Faculty of Pharmacy, Al-Zaytoonah University of Jordan, Amman 11733, Jordan; § Department of Computer Science, Faculty of Computer and Information Technology, Jordan University of Science and Technology, Irbid 22110, Jordan; ∥ Instituto de Química Médica, Consejo Superior de Investigaciones Científicas (IQM-CSIC), Madrid 28006, Spain

## Abstract

The complex and multifactorial nature of different neurodegenerative disorders hampers the capacity to identify effective treatments. Therefore, instead of relying solely on monotherapies or combination therapies, which typically come with dosing complications and limited synergy, multitarget-directed ligand strategies have emerged as one of the most dynamic and promising approaches to improve outcomes for such diseases. This study sought to identify dual modulators that specifically target cannabinoid receptor type 2 (CB2R) and muscarinic acetylcholine receptor subtype 1 (M1R), two receptors involved in various physiological and neurological processes and frequently implicated in disorders like Alzheimer’s, Parkinson’s, and chronic pain. Herein, we utilized a comprehensive computational pipeline starting with a network pharmacology analysis to map the pharmacological landscape of the dual-targeted ligands. Thereafter, molecular descriptors were employed to uncover structural similarities between CB2R agonists and M1R-positive allosteric modulators. Promising candidates were further evaluated for their binding affinities to the corresponding receptors by molecular docking studies. Collectively, these integrated computational approaches yielded a shortlist of chemotypes with the potential for dual regulation of CB2R and M1R. These findings provide a computational foundation and potential chemical starting points for future experimental studies aimed at exploring CB2R–M1R dual modulation in intricate neurodegenerative disorders and related conditions.

## Graphical abstract

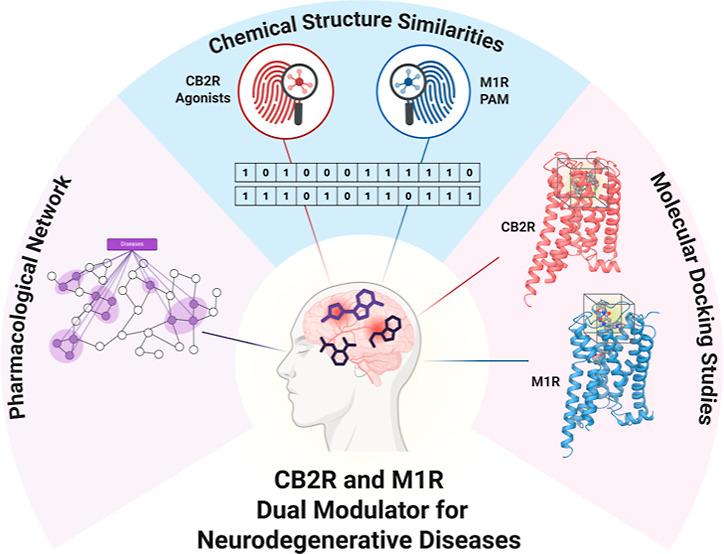

## Introduction

The intricate and multifactorial origins of various neurodegenerative disorders diminish the likelihood of identifying suitable treatments.^1^ These disorders often involve a convergence of complex molecular pathways, cellular dysfunctions, and widespread neuroinflammation, which collectively diminish the efficacy of conventional treatment strategies.^1,2^ Consequently, reliance solely on traditional therapeutic approaches, such as monotherapies or combination therapies, often proves inadequate due to suboptimal dosing regimens, adverse drug interactions, and insufficient synergy.^3^ In light of these limitations, multitarget directed ligands (MTDLs) have emerged as a promising therapeutic strategy. MTDLs can offer synergistic effects, reduce side effects, and improve pharmacokinetic profiles by simultaneously addressing numerous critical pathways related to the same disease, in contrast to single-target approaches.^4–6^ This strategy could tackle the multifaceted nature of neurodegenerative diseases more holistically and effectively.^2,7,8^

This study focuses on the computational identification and rational design of dual modulators targeting two prominent G-protein-coupled receptors (GPCRs): the cannabinoid receptor type 2 (CB2R) and the muscarinic acetylcholine receptor subtype 1 (M1R). Both receptors are critically involved in various physiological and neurological processes and frequently implicated in disorders like Alzheimer’s, Parkinson’s, and chronic pain.^9–14^

Alzheimer’s Disease (AD) is the leading cause of dementia among older patients, affecting more than 35 million people worldwide.^15^ The disease is marked by a highly multifactorial and complex pathophysiology involving numerous etiopathogenic mechanisms, such as the abnormal buildup of β-amyloid plaques and neurofibrillary tangles.^15^ In addition to disruptions across multiple neurotransmitter systems, in particular the endocannabinoid and cholinergic systems are notably impaired during AD progression.^9,11,16^

The endocannabinoid system, in particular CB2R, has been closely linked to AD. CB2R, which is predominantly expressed in the central nervous system, is significantly upregulated in AD patients and shows a positive correlation with β-amyloid plaque accumulation.^9–11^ Activation of CB2R has demonstrated a reduction in AD pathogenesis by mitigating neuroinflammation and neurodegeneration^17^ (Figure 1A). For instance, the CB2R agonist JWH-133 proved its capability to improve memory and augment the brain’s intrinsic repair mechanisms in AD rat models (Figure 1B).^18^ AM1241 nanoparticles, another CB2R agonist, efficiently mitigated neurodegenerative pathology and promoted forebrain neurogenesis in AD mouse models, collectively restoring learning and memory capabilities.^19^

**Figure 1:**
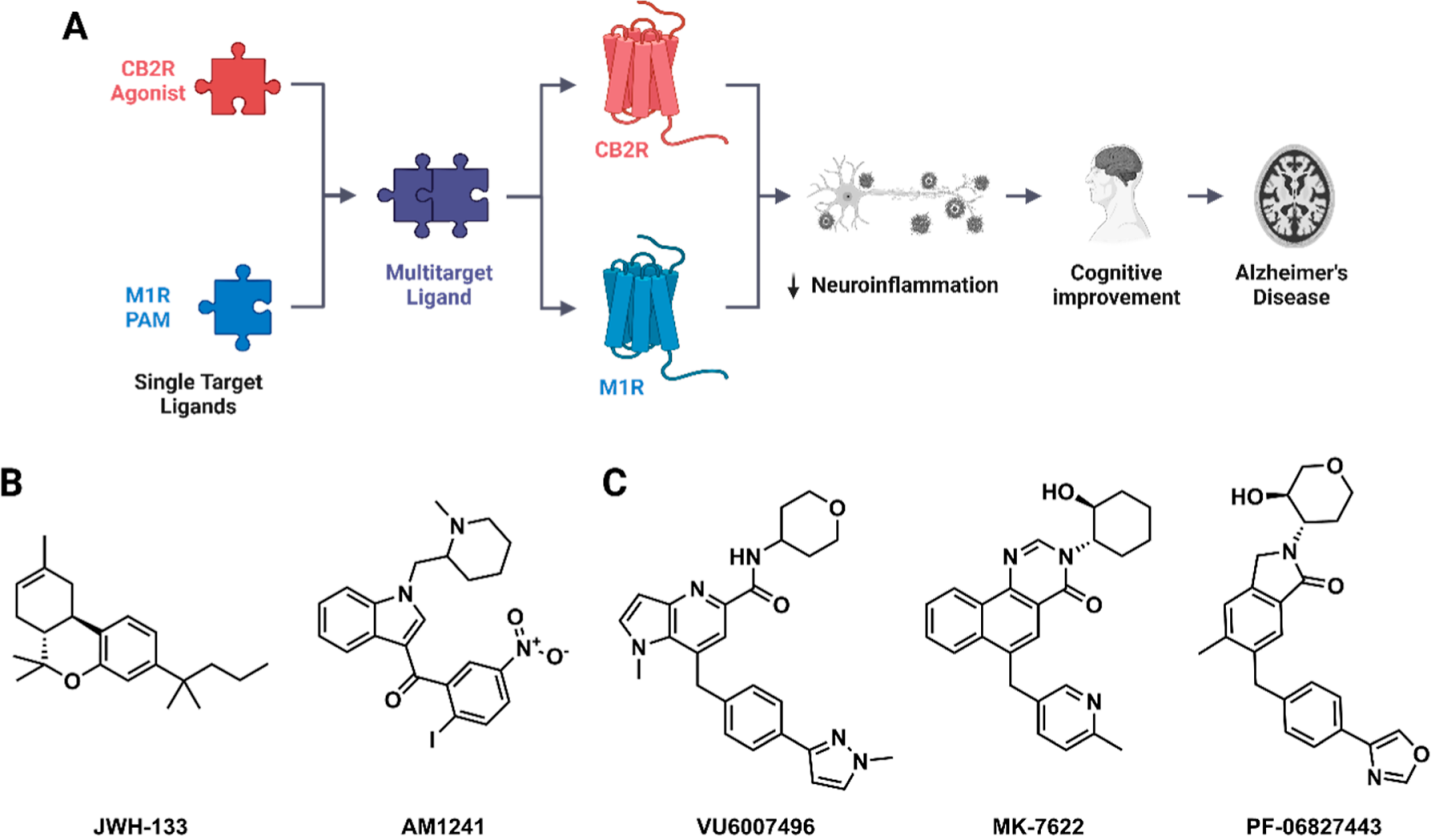
Design and mechanism of multitarget ligands targeting CB2R and M1R for Alzheimer’s Disease. (A) Schematic representation of the dual-target strategy, illustrating modulation of CB2R agonist and M1R PAM pathways in Alzheimer’s disease management. (B) Representative chemical structures of CB2R agonists. (C) Representative chemical structures of M1R PAMs. Created in BioRender*.* Isawi, I. (2026) https://BioRender.com/15n0uqd

Similarly, the cholinergic system plays a vital role in AD. According to the cholinergic hypothesis, the imbalance of acetylcholine content contributes to the cognitive decline in AD patients.^20–22^ Among the muscarinic receptor subtypes, M1R has emerged as a particularly promising route due to its ability to modulate cholinergic neurotransmission.^16,23,24^ However, the high structural similarity across all five muscarinic receptor subtypes at the orthosteric binding site makes the identification of selective M1R agonists challenging, and the lack of selectivity would be paid off as adverse effects.^16,25^ As a result, targeting allosteric sites has become an innovative approach to attain receptor subtype selectivity.^16,26,27^ In AD animal models, selective M1R-positive allosteric modulators (PAMs) have been shown not only to alleviate cognitive dysfunction but also to attenuate AD pathogenesis through the modulation of neuroinflammation and neurodegeneration (Figure 1A).^28,29^ Notably, pure M1R PAMs, such as VU6007496, demonstrated superior functional engagement compared to M1R Ago-PAMs like MK-7622 and PF-06827443, resulting in cognitive enhancement without significant adverse events (Figure 1C).^16,30^

In addition to each receptor’s role in AD, a growing body of evidence indicates that crosstalk occurs between the endocannabinoid and cholinergic systems in AD.^31–33^ Cannabinoid receptors have been demonstrated to control cholinergic dysregulation indirectly, resulting in enhanced cognitive function in AD models. Moreover, there is evidence that anandamide, an endocannabinoid fatty acid neurotransmitter, can act on both targets, functioning as a CB2R agonist and M1R PAM.^34–37^ A separate investigation indicated that the dual role of anandamide may underlie the noted improvements in cognitive performance and the attenuation in the onset of AD markers in experimental models.^38^

In light of the growing interest in MTDLs as next-generation therapies,^7^ dual ligands that can concurrently modulate CB2R and M1R represent a novel and compelling approach. Such modulators could harness synergistic effects across two dysregulated neurotransmitter systems, potentially offering novel therapeutic outcomes desirable for treating specific neurodegenerative disorders, including AD, Parkinson’s disease, and pain-related disorders.^12–14^ However, given that no small molecule has yet been experimentally confirmed to act as a dual CB2R agonist and M1R PAM, a computational exploration of their shared chemical space can provide a valuable preliminary framework for future discovery. While numerous CB2R agonist chemotypes have been recorded,^39,40^ selective M1R PAMs remain comparatively scarce.^13,16,26^

Accordingly, this work presents an integrative computational pipeline that combines multiple complementary strategies to identify and prioritize potential dual CB2R–M1R modulators (Figure 2). The framework is designed as a methodological and data resource to support future experimental and computational investigations of CB2R–M1R multitarget modulation. The workflow began with a network pharmacology analysis to map the molecular interactions of dual-target ligands with relative proteins and genes, thereby supporting the therapeutic rationale for the concurrent modulation of both receptors. Principal component analysis (PCA) was then used to identify the overlapping chemical space between CB2R agonists and M1R PAMs. This was followed by molecular descriptor analysis to detect structural similarities, utilizing molecular fingerprints (FPs) that have demonstrated effectiveness in analogous cheminformatics tasks.^41–43^ Finally, the most promising dual hits were subjected to structure-based virtual screening through molecular docking against both CB2R and M1R to evaluate their binding affinity and potential dual-target activity.

**Figure 2:**
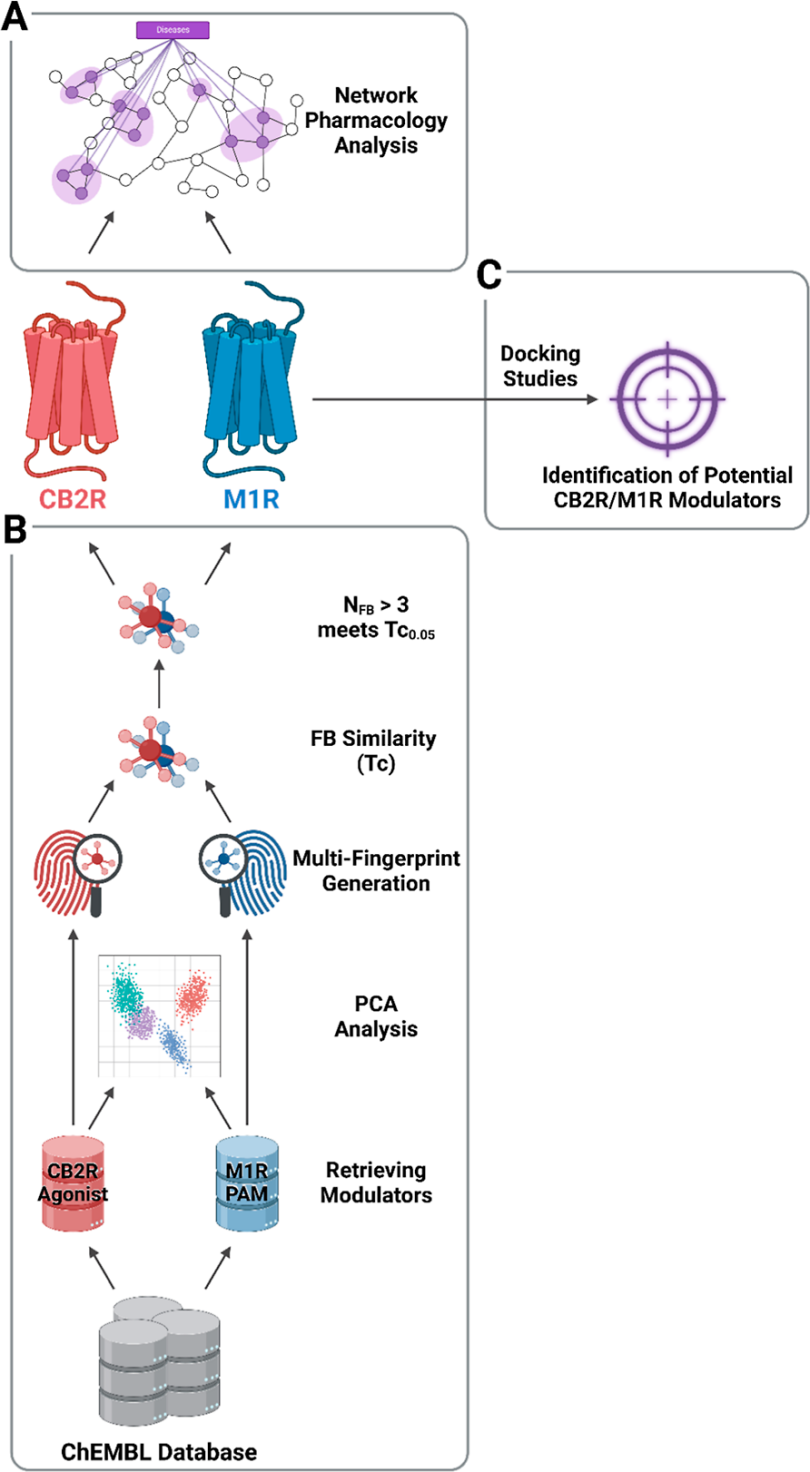
Computational pipeline for identifying CB2R-M1R dual modulators. (A) Investigating the pharmacological network of CB2R and M1R. (B) Molecular descriptor analysis to pinpoint structural similarities between CB2R agonists and M1R PAMs. (C) Docking-based screening of the top dual-target candidates against both receptors. *Created in BioRender. Isawi, I.* (2026) https://BioRender.com/15n0uqd.

Collectively, this integrative and reproducible computational workflow provides a foundation and a reusable resource for the discovery of CB2R-M1R dual chemotypes. The results yield testable hypotheses and prioritized hit candidates that can inform future experimental validation and guide the subsequent design of multitarget-directed ligands relevant to the multifactorial pathophysiology of AD and related disorders.

## Methods

### Network Pharmacology Approach

An informatics workflow was applied to study the network pharmacology of CB2R agonists and M1R PAMs, based on the methods developed by Hajjo et al.^44–46^ to formulate testable hypotheses regarding the putative mechanisms of action of these modulators. This workflow consists of two major components: (1) a network-building module to identify nearest neighbor proteins that have direct interactions with CB2R and M1R; and (2) a pathway enrichment module to elucidate the biological processes involved in the mechanism of action of the chemical compounds targeting CB2R and M1R.

### Protein–Protein Interactions

A systematic search for the nearest neighbor (NN) genes/proteins of CB2R and M1R was conducted in Cytoscape^47^ (version 3.10.3), using the STRING^48^ protein query application. All retrieved protein–protein interactions (PPIs), including both physical and functional interactions, were retrieved, and then, the network building tools in Cytoscape were utilized to generate PPI networks for CB2R and M1R.

### Enrichment Analysis

Pathway enrichment analyses for CB2R and M1R interaction networks were performed using the STRING functional enrichment tool in Cytoscape^47^ (version 3.10.3). The significance of each enrichment result was assessed by calculating the hypergeometric *p*-values. Next, all ontology terms were ranked according to their calculated false discovery rates (FDRs). The FDR is a multiple testing correction method used to control the expected proportion of false positives among the list of significantly enriched pathways. Lower FDR values indicate higher statistical confidence in the enrichment result. Herein, pathways with FDR values ≤0.05 were considered statistically significant.

### Generation and Curation of the CB2R and M1R Databases

Data were extracted from ChEMBL v34,^49^ based on the allocated ID for CB2R (CHEMBL253) and M1R (CHEMBL216). The database was filtered to exclusively incorporate entries that satisfied the following criteria: (1) assays were performed on human targets, and the target type was a single protein; (2) the assay description indicated that the compound exhibited either agonistic or partial agonistic effects on CB2R, or positive allosteric activity on M1R; and (3) there were no warnings in the “data validity comment” field. For entries with several records that existed for a target–ligand pair, only one assay type was retained for each compound. For compounds reported under the same assay but with different values, the mean value was calculated and used.

For CB2R, only compounds displaying EC_50_ ≤500 nM were retained. For M1R, compounds were retained if they displayed either an EC_50_ or IP ≤1000 nM or a positive log­(αβ) value. As a result, 2231 compounds were identified for CB2R and 442 compounds identified for M1R. The complete data set is available in the supplementary files.

The LigPrep module in Schrödinger was used to curate the retrieved SMILES by performing salt stripping and generating both the neutral and dominant ionization states of the compounds at pH 7.4, while retaining the specified chirality and varying the undefined ones.^50^ Subsequently, canonical SMILES were generated using the RDKit Canon SMILES node available in KNIME.^51^

For docking studies, compound preparation involved LigPrep (Schrödinger) with parameters set to generate potential tautomeric and ionization states at pH 7.4 ± 0.2, employing the Epik tool in classic mode.^50,52,53^ Conformer generation was performed using the ConfGen module, with up to 64 conformations per ligand, followed by energy minimization using the OPLS4 force field.^54,55^

### Principal Component Analysis

A principal component analysis was performed on a merged data set comprising all retrieved CB2R agonists and M1R PAMs. Prior to PCA, MACCS structural keys were generated for each compound, and pairwise Tanimoto similarity scores were computed. This analysis relied on the following Python libraries: NumPy, Matplotlib, RDKit, Scikit-learn, and StandardScaler.

### Fingerprint Generation

A total of 13 distinct FPs were generated for each molecule using the CDK,^56,57^ RDKit,^58^ and Pybel^59^ packages. Table S1 presents a concise overview of the computed FPs. This task was performed using the KNIME platform,^60^ utilizing its available FP nodes for CDK- and RDKit-based FPs. For FPs not supported in KNIME, custom Python scripts were used to generate fingerprints; the cleaned scripts and instructions are listed in the Data and Code Availability section.

### Molecular Similarity Calculation

The Tanimoto coefficient (*T*_c_) similarity metric was used to compute the FP-based similarity between CB2R and M1R compounds for each of the 13 distinct FP types. Two compounds were deemed similar and selected for subsequent analysis if they met the precalculated similarity thresholds, *T*_c0.05_,^41^ in at least three different FP types.

### Protein Selection and Preparation

The initial atomic coordination of CB2R-G_αi_ (PDB IDs: 6PT0, 3.20 Å;^61^ and 8GUR, 2.84 Å^62^) and M1R-G_11_ (PDB ID: 6OIJ, 3.30 Å^63^) were retrieved from the Protein Data Bank. A one-point mutation in M1R (Q110N^3.37^) (Ballesteros-Weinstein numbering in the superscript) was reverted to the wild-type sequence. The G-protein complexes were removed from both CB2R and M1R prior to applying the Protein Preparation Wizard protocol in Schrödinger, which included adding missing side chains and assigning protonation states.

### Docking Sites and Molecular Docking Studies

The docking grid for the CB2R orthosteric site was generated based on the centroid of the agonist-bound active CB2R structure (WIN 55,212–2 in PDB ID: 6PT0 ^61^ or CP 55,940 in PDB ID: 8GUR^62^). For the M1R allosteric site, the grid was centered on key amino acid residues previously identified in the literature as critical for PAM interactions, along with additional residues that comprehensively cover the relevant extracellular region. These residues include Y85^2.63^, T172^ECL2^, Q177^ECL2^, Y179^ECL2^, L183^ECL2^, K392^ECL3^, E393^ECL3^, E397^ECL3^, W400^7.35^, and E401^7.36^. A 20 Å cubic docking grid was applied to both targets. All crystallographic water molecules were removed, and no water was retained in the docking grids. Additionally, no constraints, rotatable side chains, or excluded volumes were applied to either grid.

Schrödinger’s Induced Fit Docking (IFD) protocol was employed to accommodate receptor flexibility during ligand docking in both CB2R and M1R. For CB2R, both agonist-bound structures were used for cross-docking to explore ligand binding across two distinct ligand scaffolds—WIN 55,212–2 and CP 55,940. In the initial Glide docking step, the van der Waals scaling factor was set to 0.6 for both the receptor and the ligand. The Prime refinement stage was applied to the side chains of residues within 5 Å of the ligand. For each ligand, a maximum of 10 poses were retained for subsequent redocking in the extra precision (XP) mode.

### Visualization and Figure Preparation

Schematic illustrations and graphical representations were created with BioRender (Figures 1 and 2) under a BioRender Publication License. Figure 3 was generated using Cytoscape (v3.10.3),^47^ and Figure 4 was produced with Python/Matplotlib (*matplotlib.pyplot*, v3.10.0).^64^ All compounds retrieved from ChEMBL were redrawn and standardized in ChemDraw prior to docking (Figures 6 and 8). All molecular docking visualizations and structural renders were generated using the Schrödinger Suite (Release 2025–2) (Figures 5, 7, 9, S2 and S3).

**Figure 3:**
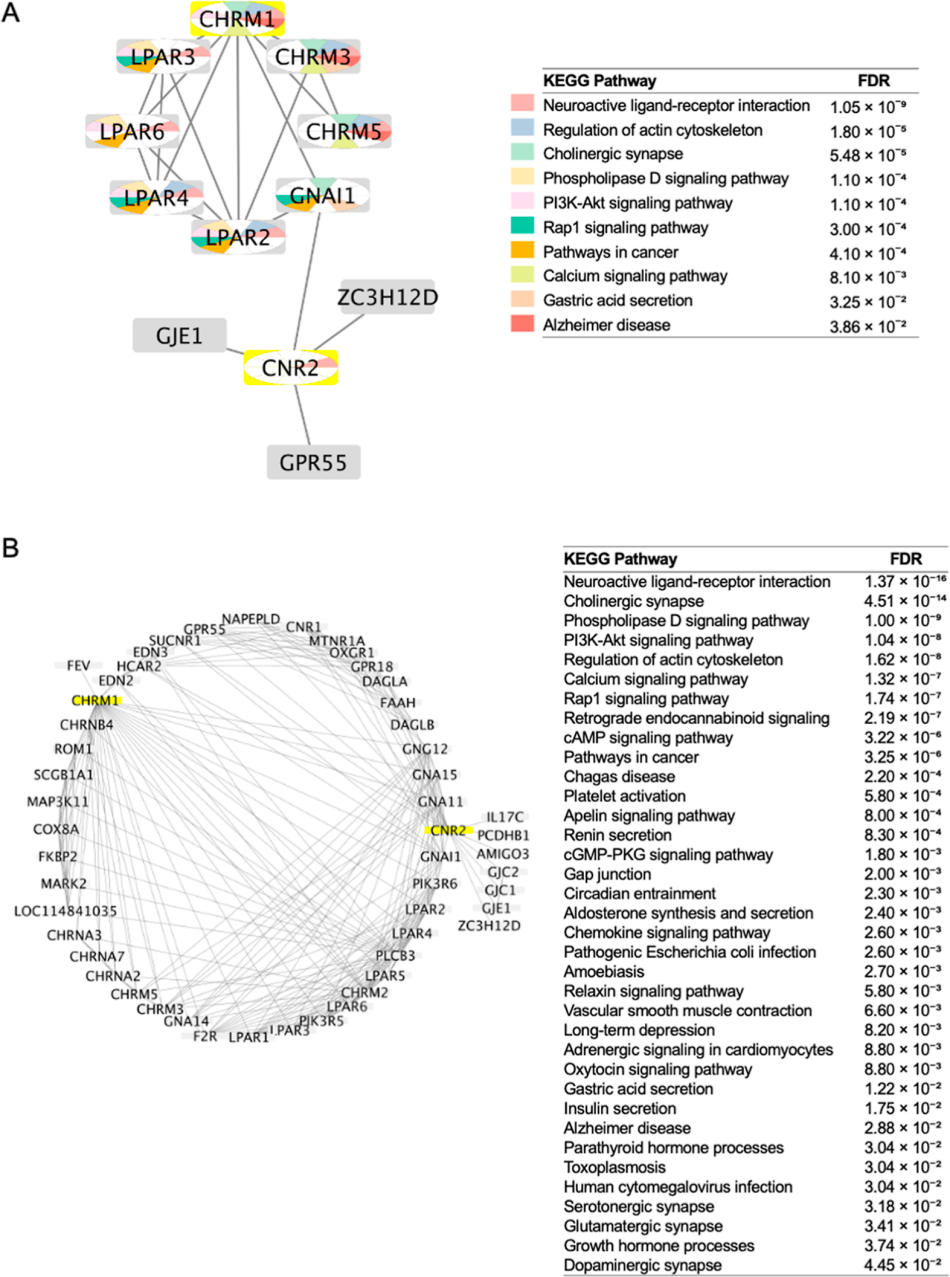
Downstream signaling networks and interacting protein partners of CB2R and M1R. (A) Network expanded to include the 10 nearest interacting proteins for each receptor. (B) Network expanded to include the 50 nearest interacting proteins. In both panels, the original query proteins (CB2R and M1R) are highlighted in yellow, while all other nodes are shown in gray.

**Figure 4:**
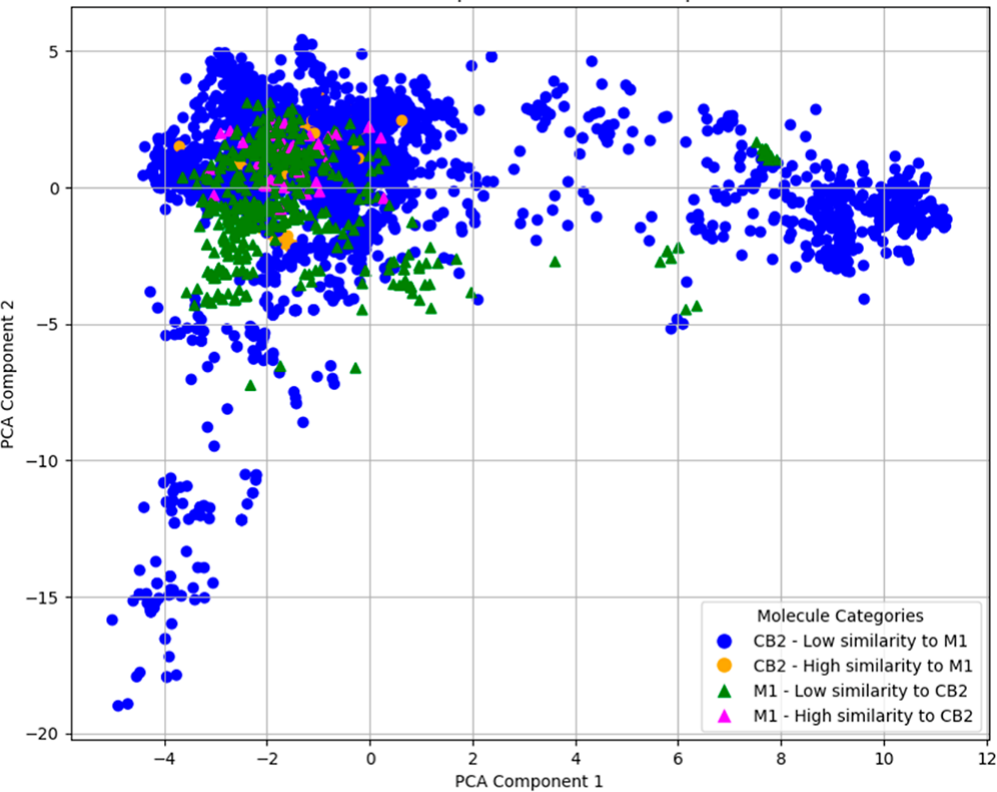
PCA of molecules from CB2R and M1R based on the MACCS keys. Orange circles and magenta triangles indicate ligands that share high similarities with the ligands from the other class. High similarity in this figure indicates a Tanimoto similarity of ≥0.80, while low similarity indicates a Tanimoto similarity of ≤0.80.

**Figure 5:**
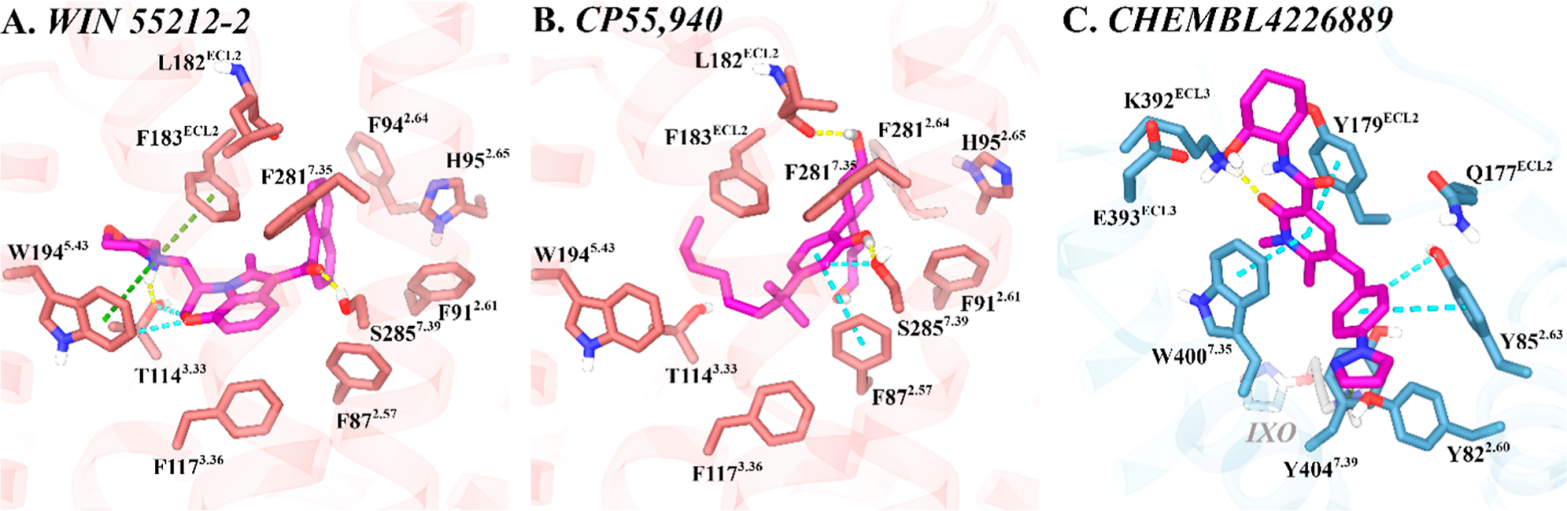
CB2R orthosteric binding site and M1R allosteric binding site. (A) WIN 55212–2 and key binding residues in CB2R (PDB ID: 6PT0). (B) CP 55,940 and key binding residues in CB2R (PDB ID: 8GUR). (C) CHEMBL4226889 and key binding residues in the proposed M1R allosteric site in the presence of the orthosteric agonist Iperoxo (IXO) (PDB ID: 6OIJ). CB2R and M1R are shown as rose and sky-blue ribbons, respectively. Key ligand-binding residues are displayed as dark rose and dark sky-blue sticks, respectively. Ligands are represented as magenta sticks within their respective binding sites. Hydrogen bonds are indicated with yellow dashed lines, π–π-stacking interactions with cyan dashed lines, aromatic hydrogen bonds with light cyan dashed lines, and π–cation interactions with green dashed lines.

**Figure 6:**
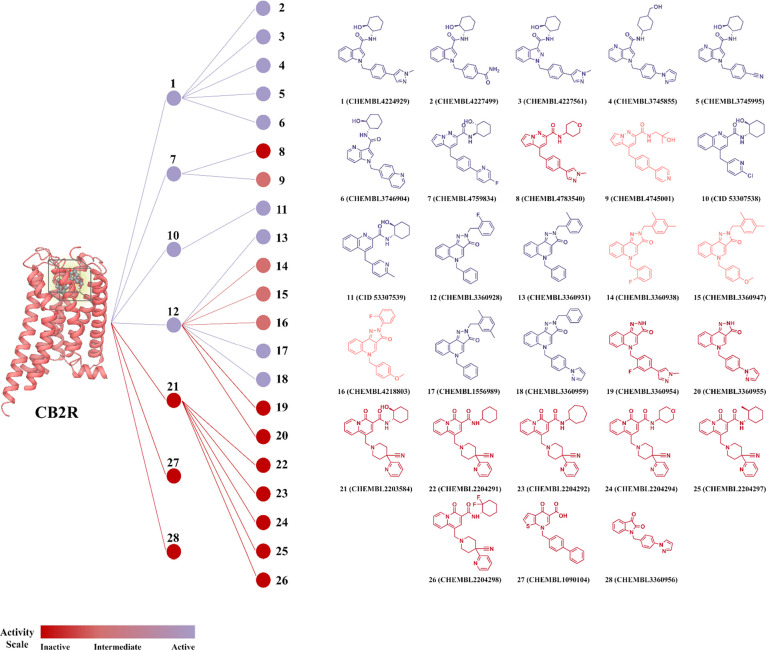
Predicted affinity/binding distribution of M1R PAM compounds in the CB2R orthosteric binding site by scaffold-defined groups. (A) color-coded tree map illustrates the distribution of predicted binding affinities for the M1R PAM compounds at the CB2R orthosteric binding site, grouped according to shared chemical scaffolds.

**Figure 7:**
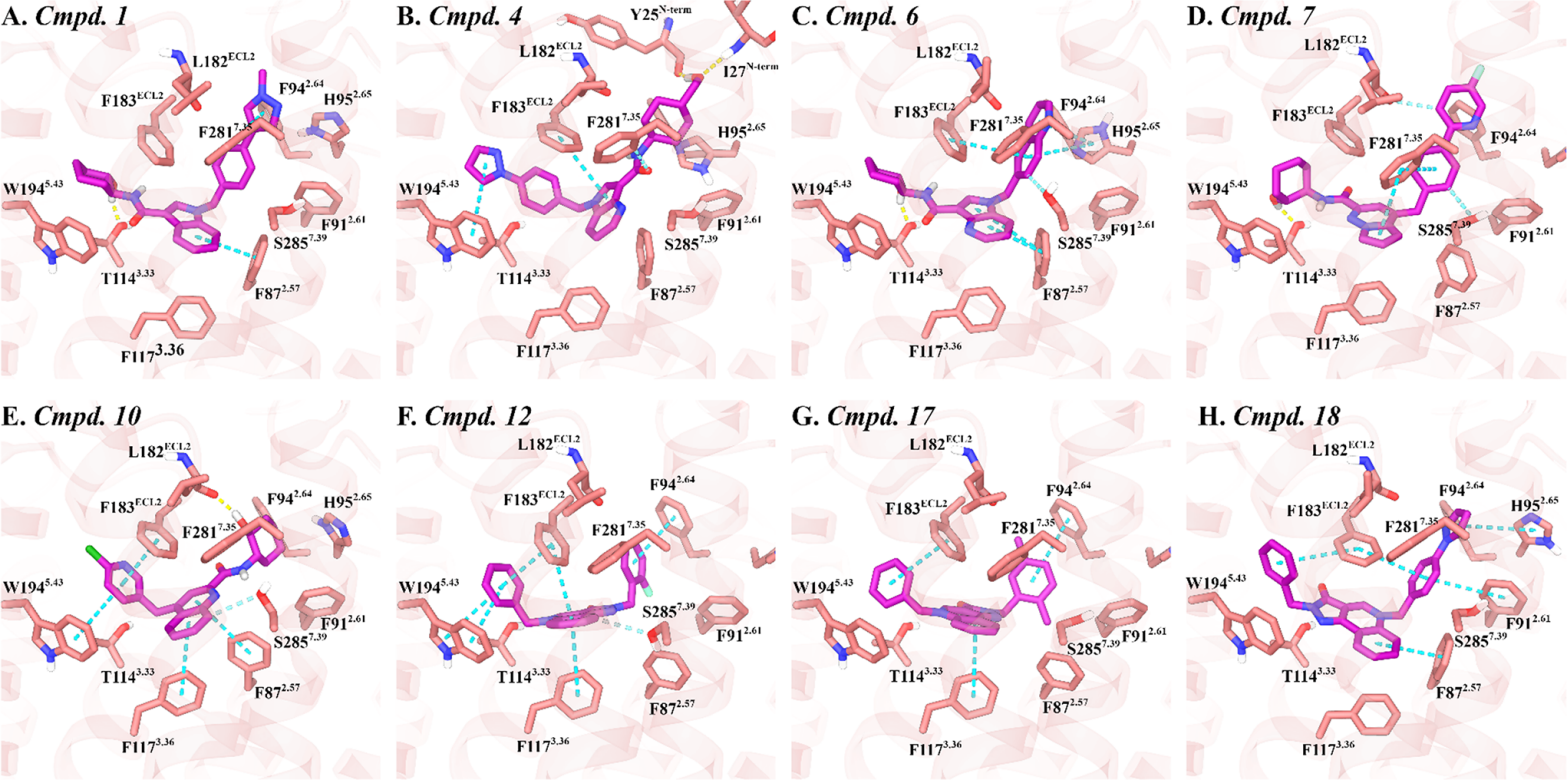
Key interactions between the M1R PAMs docked within the CB2R orthosteric binding pocket. Key interacting residues for (A) Compound **1**, (B) Compound **4**, (C) Compound **6**, (D) Compound **7**, (E) Compound **10**, (F) Compound **12**, (G) Compound **17**, and (H) Compound **17**, all docked into the orthosteric site of CB2R (PDB ID: 8GUR or 6PT0). Color coding is consistent with that in Figure 5

**Figure 8:**
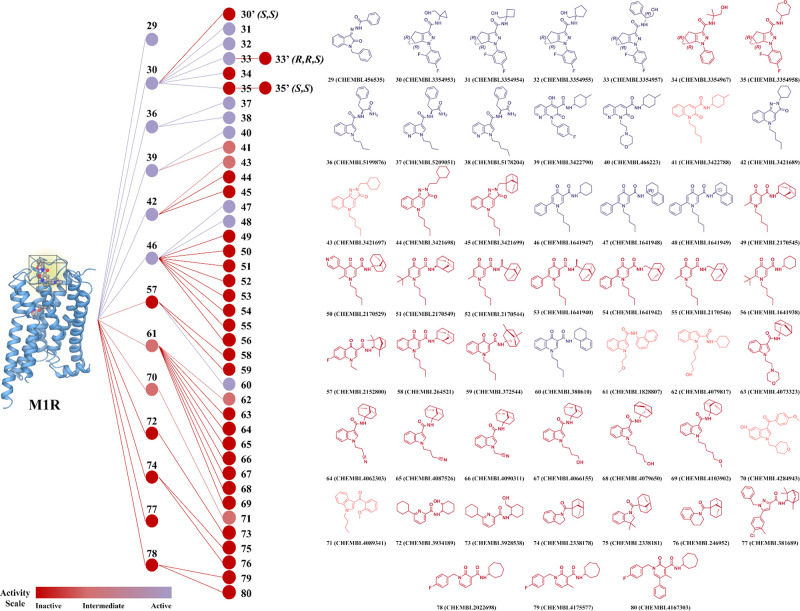
Predicted affinity/binding distribution of CB2R agonist compounds in the M1R allosteric binding site by Scaffold-Defined Groups. (A) color-coded tree map illustrates the distribution of predicted binding affinities for CB2R agonist compounds within the M1R allosteric binding site, grouped according to shared chemical scaffolds.

**Figure 9:**
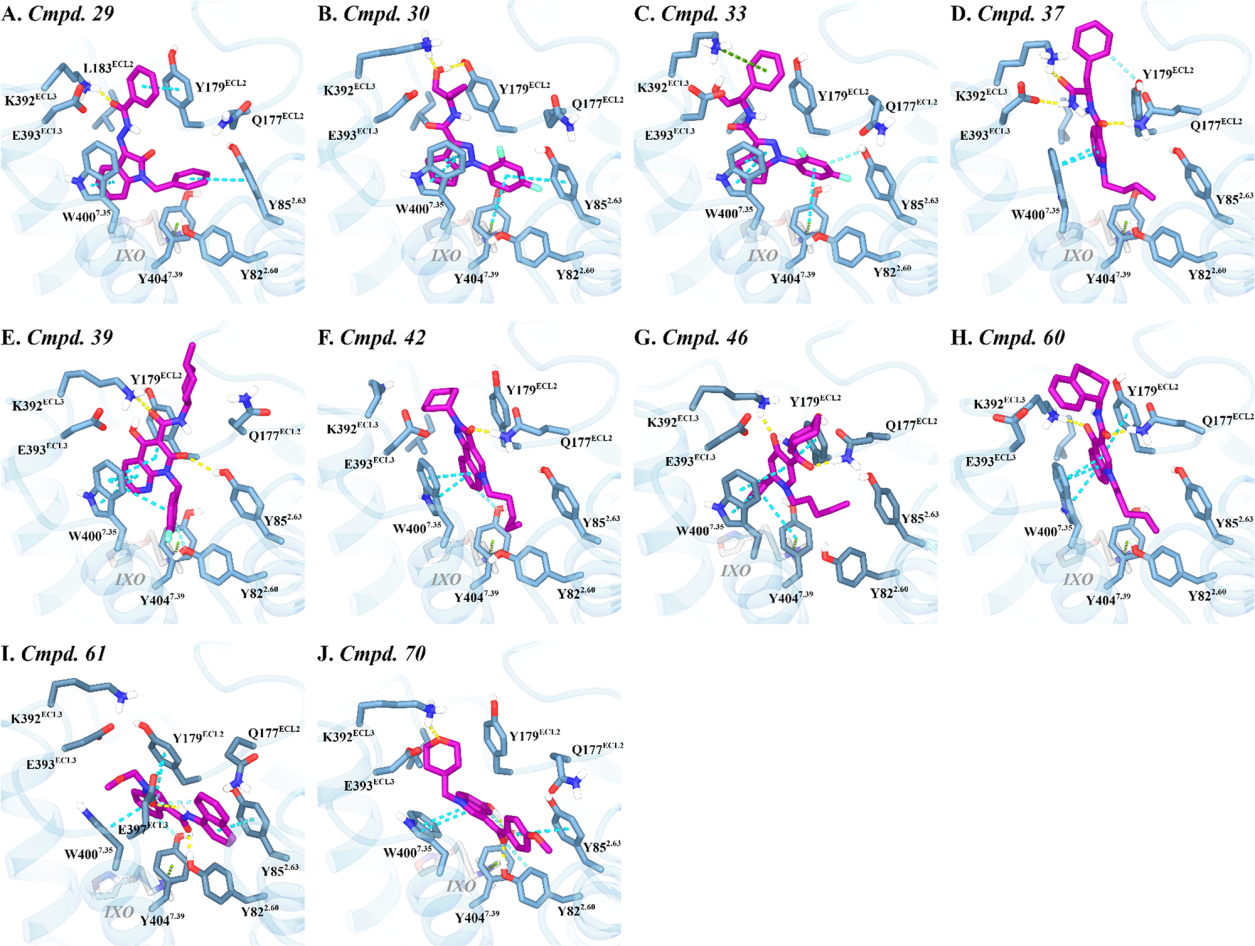
Key interactions between the CB2R agonists docked within the M1R allosteric binding pocket. Key interacting residues for (A) Compound **29**, (B) Compound **30**, (C) Compound **33**, (D) Compound **37**, (E) Compound **39**, (F) Compound **42**, (G) Compound **46**, (H) Compound **60**, (I) Compound **61**, and (J) Compound **70**, all docked into the allosteric site of M1R in the presence of the orthosteric agonist Iperoxo (IXO) (PDB ID: 6OIJ). Color coding is consistent with Figure 5

## Results and Discussion

### Workflow for Identifying Dual Ligands for CB2R and M1R

In developing MTDLs, two key steps are essential before progressing further.^65^ First, the targets of interest must act synergistically to modulate pathological processes and enhance therapeutic outcomes—an approach that builds on the independent effectiveness of each target against the same pathology. Second, modulators are required to exhibit either overlapping chemical properties or similar binding pocket characteristics. In this work, given the broad therapeutic applications of both CB2R and M1R across an array of diseases, we initially performed a pharmacology network analysis to elucidate the disease association of these receptors with particular interest toward neurodegenerative disorders (Figure 2A). Then, a PCA was conducted to evaluate the shared chemical space between CB2R agonists and M1R PAMs (Figure 2B). This type of analysis was previously successfully used as a preliminary step in identifying MTDLs, including those that combine CB2R modulation with targets such as histone deacetylases (HDAC), sigma-1 receptor,^66^ and fatty acid amide hydrolase (FAAH^67^).^67^ Encouraged by the network pharmacology and PCA findings, we then employed a multifingerprint consensus strategy utilizing 13 distinct FP types to capture independent spatial dimensions and improve the confidence of our similarity predictions (Figure 2B).^41–43^ Finally, leveraging available structural data of both GPCRs in complex with their corresponding G proteins,^63,68^ structure-based docking studies were performed to identify the most promising dual-target hits (Figure 2C).

### Pharmacology Network Analysis to Assess Biological Similarity

To investigate the potential biological relevance of the CB2R and M1R dual modulators, we conducted an integrated pharmacological network analysis. Initially, a PPI network was constructed using CB2R and M1R as seed nodes, expanded by their 10 nearest interacting partners using the STRING protein query tool within Cytoscape (version 3.10.3), as shown in Figure 3A. Functional enrichment analysis of the resulting network was performed using the STRING Enrichment app in Cytoscape, revealing several significantly enriched Kyoto Encyclopedia of Genes and Genomes (KEGG) pathways. The top pathways included neuroactive ligand–receptor interaction, regulation of actin cytoskeleton, cholinergic synapse, phospholipase D signaling pathway, PI3K-Akt signaling pathway, Rap1 signaling pathway, pathways in cancer, calcium signaling pathway, gastric acid secretion, and AD.

To explore a broader signaling context, the network was subsequently expanded to include 50 additional interacting proteins (Figure 3B). This larger network yielded enrichment of 36 KEGG pathways, with top hits again featuring neuroactive ligand–receptor interaction, cholinergic synapse, phospholipase D signaling, PI3K-Akt signaling, regulation of actin cytoskeleton, calcium signaling, Rap1 signaling, retrograde endocannabinoid signaling, and cAMP signaling. AD remained among the enriched pathways, though it was ranked 29th enriched with an FDR of 2.88 × 10^–2^, indicating moderate statistical significance in the broader interaction context.

### Assessing the Overall Structural Similarity between CB2R and M1R Ligands

In order to assess the feasibility of designing dual ligands that interact with both CB2R and M1R receptors, it is crucial to understand how similar the ligands are between the CB2R agonists and the M1 PAM. Ligand similarity assessment helps determine how molecules interact with both receptors and their potential for cross-target binding and may even provide further insights into their functional properties. Herein, MACCS keys—166-bit structural fingerprints encoding the presence or absence of predefined substructures—were generated as FPs for all ligands known to interact independently with CB2R and M1R. These fingerprints are widely used for their balance of interpretability and efficiency and have been shown to capture key structural—and potentially functional—properties of molecules, as previously described by Hajjo et al.^69,70^ PCA was then performed on the MACCS key representations to explore the chemical space and assess the similarity between the two ligand groups.

The PCA plot shown in Figure 4 provides a clear visual representation of the ligand similarities between CB2R and M1R. The clustering of ligands in the PCA plot indicates how similar (i.e., structurally related) or dissimilar (i.e., structurally distinct) the molecules are based on their MACCS FPs. Clearly, there is a good proportion of CB2R ligands that cluster together with M1R ligands in the same region of the plot. In fact, 2701 CB2R ligands spread over a large proportion of the multidimensional space formed by 166 MACCS keys and have an average Tanimoto similarity of 0.47, while the 626 M1R ligands were less dispersed, which could be due to the smaller data set size, and they had an average Tanimoto similarity of 0.58. However, the average Tanimoto similarity between the CB2R and M1R ligands was 0.45.

Thus, CB2R and M1R modulators that clustered together have similar chemical structures and, therefore, may exhibit similar binding profiles and biological activities toward both receptors. Conversely, ligands that are spread out in the plot, occupying distinct regions, suggest significant structural differences, indicating that they may interact with the designated receptors in fundamentally different ways.

Figure 4 highlights regions of the PCA plot where the ligands from CB2R and M1R overlap and share an overall structural similarity of ≥0.80, indicated by the orange circles for CB2R ligands and magenta triangles for overlapping M1R ligands. These overlaps with high Tanimoto similarities suggest potential cross-reactivity toward both CB2R and M1R receptors. This analysis resulted in 77 CB2R ligands that share ≥0.80 Tanimoto similarity with M1R ligands and 57 M1R ligands that share ≥0.80 Tanimoto similarity with CB2R ligands (Table 1).

**Table 1:**
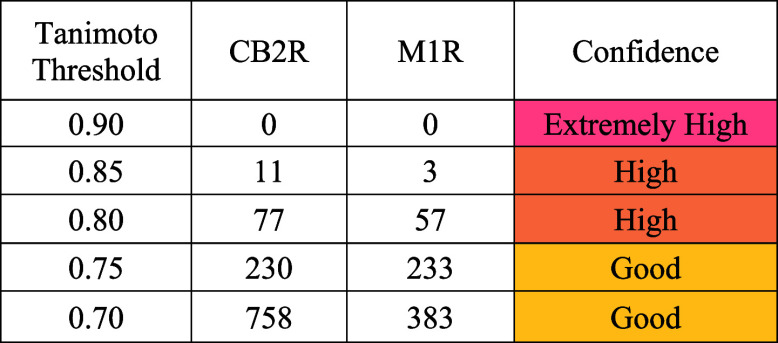
Numbers of Retrieved CB2R and M1R Ligands That Could Potentially be Dual Inhibitors for Both Receptors

### Multi-Fingerprint Consensus Analysis and Selectivity Filtering for Compound Prioritization

Molecular descriptors are a suitable choice for capturing similarities between compound groups, with molecular FPs being among the most widely used and effective tools in this context. For example, the Nicolotti group developed an effective approach for predicting drug targets and bioactivity using the Multifingerprint Similarity Search Algorithm.^41–43^ In our study, a similar consensus-based strategy was employed, incorporating 13 distinct FPs, spanning structural, circular, and topological descriptors (Table S1, Supplementary file). These FPs exhibit low intrinsic correlation with one another, indicating that each represents a unique region of chemical space.^41^ By leveraging this diversity, the approach aims to capture independent spatial dimensions of molecular similarity, thereby improving the robustness and reliability of similarity-based predictions.

Similarity values between each CB2R agonist and M1R PAM were computed using the *T*_c_. While the “0.85 Myth” posits that compounds with *T*_c_ values of 0.85 or higher frequently demonstrate similar biological activity,^71^ this fixed threshold may not necessarily account for the subtle variations among FPs, as certain FPs emphasize more prevalent features, while others highlight fewer common features. To address this limitation, the Nicolotti group proposed a statistically derived similarity threshold at the 5% significance level (*T*_c0.05_), based on 10 million random pairwise comparisons from ChEMBL.^41^ Applying this criterion, 67 unique compounds from the CB2R agonist set and 33 unique compounds from the M1R PAM set met the *T*_c0.05_ threshold in at least three distinct FP types.

Subsequently, these smaller sets were re-evaluated for selectivity to ensure the absence of reported activity against known off-targets. This step aims to mitigate the risk of undesirable pharmacological events in the future. The selectivity screening was guided by data from PubChem,^72^ leading to the exclusion of promiscuous compounds with multitarget activity, particularly those exhibiting a selectivity window of less than 10-fold.^73^ Meanwhile, compounds with reported affinity for *P*-glycoprotein (*P*-gp) efflux transporters were not excluded, as they may still hold relevance for non-CNS indications, despite the study’s primary CNS focus. Consequently, 56 of the original 67 CB2R agonists and 28 of the 33 M1R PAMs were retained for further evaluation. The final selected compounds are listed in Tables S2 and S3, respectively (Supplementary file).

### In Silico Investigation of Cross-Affinity Among Prioritized Compounds

#### CB2R-Orthosteric and M1R-PAM Binding Sites

Experimental structures of both CB2R and M1R were used to construct the in silico docking models, with known modulators serving as guides for optimizing docking parameters. To contextualize the subsequent discussion on binding analysis, key interaction patterns and residues involved in ligand recognition for each receptor are summarized below.

In the last years, the conformational space of CB2R has been experimentally elucidated in both its active and inactive states.^61,62,74,75^ Recently released cryo-EM structures of CB2R in complex with G_i_ protein demonstrate that the orthosteric binding pocket—targeted by well-known agonists such as HU 308,^62^ WIN 55212–2,^61^ and CP 55,940 ^62^—is primarily formed by residues from transmembrane helices (TMHs) 2, 3, 5, and 7 and is capped by the second extracellular loop (ECL2). Key residues involved in ligand recognition include F87^2.57^, F91^2.61^, F94^2.64^, H95^2.65^, F106^3.25^, and F117^3.36^, which engage in strong hydrophobic and aromatic interactions, along with ECL2 residues F183^ECL2^ and P184^ECL2^ that are projected into the binding crevice (Figure 5A). Additional residues such as T114^3.33^ ^62^ and S285^7.39^ ^62,76^ are notable for their capacity to form hydrogen bonds with a range of ligands. In contrast, inactive state structures show that antagonists need to go deeper into the highly hydrophobic pocket to stabilize the toggle switch residue W258^6.48^ in its inactive rotamer, thereby locking the receptor in its inactive conformation.^74,76^

Although no structure of the M1R in complex with a PAM is currently available, valuable insights can be drawn from the muscarinic acetylcholine receptor subtype 2 (M2R) structures in complex with an orthosteric agonist and its selective PAM LY2119620 (PDB IDs: 4MQT, 3.70 Å^25^ and 6OIK, 3.60 Å^63^). These data, combined with M1R site-directed mutagenesis data,^27,77^ molecular docking, and structure-activity relationship (SAR) analyses of M1R PAMs,^78,79^ suggest that M1R PAMs such as VU6007496, MK-7622, and PF-06827443 bind to an allosteric site located above the orthosteric pocket, on the extracellular surface. This binding mode is similar to that observed for M2R PAMs, with the modulators oriented toward ECL2 and ECL3.^13^

Potent M1R PAMs tend to adopt a bent three-dimensional geometry that facilitates optimal binding. In this conformation, a hydrophilic headgroup is positioned to anchor the ligand at the mouth of the extracellular vestibule via hydrogen bonds with distinctive M1R residues such as Q177^ECL2^, K392^ECL3^, and E393^ECL3^ (Figure 1C,B). A central aromatic moiety orients the polar head and separates it from the lipophilic tail, typically forming three-layer π–π stacking interactions with W400^7.35^ and Y179^ECL2^ residues. Extending from this core is a tail composed of mono- or diaryl/heteroaryl groups projected into a lipophilic pocket formed by TMHs 2 and 7. This tail reaches into an aromatic-rich region, engaging residues such as Y82^2.60^ and Y85^2.63^, and it may also interact with negatively charged residues like E397^ECL3^, another M1R-distinctive residue, potentially stabilizing a positively charged tail.

#### Validation of Docking Methods

To assess the accuracy and reliability of our docking methodology at the CB2R, we redocked the cocrystallized ligands from the CB2R structures—WIN 55,212–2 (PDB ID: 6PT0)^61^ and CP 55,940 (PDB ID: 8GUR)^62^ into their corresponding cryo-EM structures using IFD. The resulting poses showed root mean squared deviation (RMSD) values of 1.030 Å and 0.402 Å, respectively, relative to the experimental coordinates (Figure S1), confirming that the protocol reliably reproduces experimentally determined binding modes, thus validating its suitability for subsequent docking studies at the CB2R.

For the M1R-PAM system, where no structure is being cocrystallized with a PAM, the active-state M1R structure in complex with the orthosteric agonist Iperoxo (IXO) (PDB ID: 6OIJ)^63^ was used for docking CHEMBL4226889 (a well-characterized M1R PAM with an EC_50_ of 29 nM^78^) at its allosteric site. The compound was selected for IFD due to its high potency and strong literature precedent. This compound guided model refinement and docking parameter optimization for subsequent studies. The resulting IFD-refined M1R-PAM complex produced a binding pose consistent with previously proposed models and known SAR data.^27,77^ The model accommodated the ligand with plausible binding orientations and favorable docking scores, supporting its suitability for the virtual screening of potential M1R PAM candidates.

#### Molecular Docking-Based Screening

For docking studies, compounds were first categorized based on shared scaffolds to streamline comparisons and facilitate interpretation of SAR within each scaffold class. Docking analysis was then guided by a combination of docking scores, published SAR data for each modulator family, and personal in cerebro insights derived from prior experience.

#### Docking Studies of the M1R PAMs at the CB2R Orthosteric Site

A thorough analysis of docking results for the 28 selected M1R PAMs within the orthosteric pocket of active-state CB2R revealed several compounds with favorable binding profiles, highlighting their potential as starting points for dual CB2R–M1R modulator development. Preferred binding poses were identified based on consistent interaction patterns and spatial alignment within the binding pocket.

A prominent class of active compounds features fused *N*-heterobicyclic aromatic scaffolds—such as indoles, indazoles, or pyrrolopyridines—which were notably enriched among the top hits (Compounds **1–6**, Figure 6). Docking studies indicated that these bicyclic systems are centrally positioned within the CB2R orthosteric pocket, anchored through π–π stacking with key aromatic clusters, including F87^2.57^, F117^3.36^, F183 ^ECL2^, and F281^7.35^. The five-membered ring of the cores is substituted with various para-substituted benzyl groups that extend into the cleft between TMHs 2 and 7, forming additional π–π interactions with H95^2.65^, F183 ^ECL2^, and F281^7.35^, or engaging in hydrogen bonds with L182^ECL2^ when bearing hydrophilic substituents. At the opposite end, the central scaffolds are linked to cyclohexyl carboxamide moieties, which adopt a stable chair conformation and protrude into the narrow channel between TMHs 3 and 5. This region allows for hydrogen bonding with T114^3.33^ and hydrophobic contacts with W194^5.43^—mirroring the binding orientation of the morpholine moiety in WIN 55,212–2 ^61^ and the tetrahydropyran ring in LEI-102.^62^ Among this series, compounds **1**, **2**, **3**, **5**, and **6** displayed binding poses consistent with this profile (Figures 7A,C, S2A,B,C). In contrast, compound **4**, which incorporates a bulkier hydroxymethyl-substituted cyclohexyl group, adopted an inverted binding orientation (Figure 7B). In this pose, the hydroxymethyl cyclohexyl moiety is oriented toward the *N*-term, resembling the binding mode of the cyclohexanol group in CP 55,940,^62^ while still engaging F87^2.57^, F183^ECL2^, and W194^5.43^. This chemotype thus represents a promising scaffold for dual CB2R-M1R modulation with the potential to fine–tune activity at both receptors via strategic substitutions on the central bicyclic core.

In the case of the pyrrolopyridazine scaffold (Compounds **7–9**, Figure 6), the nature of the substituents on both arms affects the compound’s ability to engage the CB2R orthosteric crevice. Compounds **7** and **9** successfully expand within the binding pocket, establishing an aromatic cluster with residues from TMHs 2, 3, and 5 (Figures 7D and S2D). In these poses, the bicyclic arm is positioned near TMHs 2 and 7 to facilitate hydrophobic interactions with F281^7.35^ or F94^2.64^, while the carboxamide headgroup is oriented toward TMHs 3 and 5, enabling hydrogen bonding with T114^3.33^.

Moreover, two quinoline derivatives—this time featuring fused six-membered aromatic rings—adopted binding poses reminiscent of the orientation observed for CP 55,940, likely due to their larger central core and shorter aromatic arms (Compounds **10–11**, Figure 6). In these poses, the hydroxycyclohexyl moiety is oriented extracellularly, positioned near TMHs 2 and 7. In compound **10**, it forms a hydrogen bond with L182^ECL2^, while in compound **11**, it interacts with S285^7.39^ (Figures 7E and S2E). The quinoline cores are stabilized through π–π stacking with F87^2.57^ and F117^3.36^, and the benzyl substituents engage with F183^ECL2^ and W194^5.43^.

Similarly, the pyrazolo quinolinone scaffold holds promise as a potential dual-acting chemotype for CB2R–M1R modulation (Compounds **12–20**, Figure 6). This tricyclic system spans the CB2R orthosteric pocket and engages in a combination of aromatic and hydrophobic interactions with residues from TMHs 2, 3, and 5, as well as ECL2. Docking studies revealed that more branched substituents on these compounds generally orient toward the cleft between TMHs 2 and 7, while smaller substituents project toward the channel between TMHs 3 and 5 (Figures 7F–H and S2F–I). Some of the most favorable binders in this series are compounds **12** and **13**, whose tricyclic cores display strong π–π stacking interactions with F117^3.36^ and F183^ECL2^ (Figures 7F and S2F). In these compounds, the fluorobenzyl (compound **12**) or methylbenzyl (compound **13**) substituents stack with F94^2.64^, while the benzyl tail engages in additional stacking with F183^ECL2^ and W194^5.43^, reinforcing their anchoring within the pocket. Compound **18** similarly demonstrates extensive aromatic contacts: the tricyclic core stacks with F87^2.57^; the pyrazolyl-benzyl arm engages F91^2.61^, H95^2.65^, and F183^ECL2^; and the benzyl group also reinforces binding via interactions with F183^ECL2^ (Figure 7H). In contrast, compounds lacking one of the arm substituents—such as **19** and **20**—display poor CB2R binding. In these cases, the central scaffold is positioned too high within the binding crevice, failing to establish the critical anchoring interactions necessary for stable binding.

On the other hand, compounds bearing a quinolizinone core—represented by M1R allosteric modulators **21** to **26**—exhibited limited ability to fit inside the CB2R (Figure 6). These molecules possess highly branched architectures that prevent effective engagement of the central aromatic core with the hydrophobic orthosteric pocket of CB2R. As a result, they display overall poor shape complementarity and suboptimal interaction patterns, leading to a reduced binding potential.

While compounds **27** and **28**—featuring thienopyridinone and indolinedione scaffolds, respectively—were found to be too small to effectively occupy the key subpockets of the CB2R binding site, further limiting their binding potential (Figure 6).

#### Docking Studies of the CB2R Agonists at the M1R Allosteric Site

Structural analysis of docking studies for the 56 selected CB2R modulators revealed several compounds with key structural characteristics compatible with the M1R allosteric binding site, indicating their potential as dual CB2R-M1R modulators.

Among these, compound **29**, featuring an oxoindolinylidene benzohydrazide scaffold, showed promising results due to its optimal bent conformation, which fits snugly within the M1R PAM binding site (Compound **29**, Figures 8 and 9A). Docking studies indicated that the oxoindolinylidene moiety aligns parallel to W400^7.35^, enabling aromatic stacking; the benzohydrazide carbonyl’s proximity to K392^ECL3^ facilitates an ion–dipole interaction, and its benzene ring engages in π–π stacking with Y179^ECL2^. Concurrently, the benzyl tail projects toward the aromatic-rich region between TMHs 2 and 7, forming additional stacking interactions that further stabilize the binding pose.

A second promising scaffold class includes the cyclopentapyrazole carboxamides, which were particularly abundant and featured structural variations at the carboxamide cap (Compounds **30–35**, Figure 8). Remarkably, the *R*,*R*-cyclopropyl isomers demonstrated superior accessibility and fit within the M1R PAM pocket, suggesting a favorable conformational bias. In this series, the pyrazole cores are stacked with W400^7.35^, which is applicable only when the cyclopropyl group is oriented away from this residue in the R,R isomers. The carboxamide head groups extended toward the extracellular vestibule and could form key hydrogen bonds with Q177^ECL2^, K392^ECL3^, and/or D393^ECL3^, and in some cases engage in π-cation interactions with K392^ECL3^ depending on the nature of the cap. Meanwhile, the difluorophenyl moiety further stabilizes the pose via stacking not only with Y85^2.63^ but also with Y404^7.39^, the latter being a residue known for its role in π-cation interactions with orthosteric agonists and potentially contributing to receptor stabilization. Among this series, compounds **30–33** emerged as the most promising derivatives (Figures 9B,C and S3A,B , respectively). In contrast, compounds featuring bulky noncyclic caps (e.g., compound **34**) or lacking branched polar groups on their cyclic caps (e.g., compound **35**) failed to establish effective interactions. Overall, the structural diversity at the cap positions provides a compelling foundation for systematic SAR exploration and offers strong potential for fine-tuning dual activity toward the development of optimized modulators.

Indole and pyrrolopyridine carboxamide derivatives also showed promising characteristics (Compounds **36–38**, Figure 8). These compounds incorporate an additional carboxamide group at the head moiety, paired with a benzylic substituent, permitting extra hydrogen bonds and potential π-cation interactions with ECL2 or ECL3 residues. Docking indicated that the indole or pyrrolopyridine carboxamide promotes aromatic stacking with W400^7.35^ and/or Y179^ECL2^, along with hydrogen bonding with Q177^ECL2^ (Figures S3C,D and 9D, respectively). The appended benzyl carboxamide could further contribute by forming additional hydrogen bonds with Q177^ECL2^, E393^ECL3^, and/or K392^ECL3^. Despite the fact that these derivatives have hydrocarbon tails rather than rigid aromatic systems, docking results suggested that the aliphatic chains are accommodated within the hydrophobic pocket between TMHs 2 and 7. This compatibility highlights their good potential for further structural optimization.

Due to their strong structural resemblance to well-known M1R PAMs, especially compound **39**, the naphthyridinone carboxamide scaffold stood out as one of the most promising dual modulators (Compounds **39–41**, Figure 8). Its bent conformation enabled the bicycle core to engage in π–π stacking with W400^7.35^ and/or Y179^ECL2^, while the carbonyl oxygens formed hydrogen bonds with K392^ECL3^ and Y85^2.63^, and the aromatic tail extended toward the TMHs 2 and 7 region (Figure 9E). Derivatives lacking aromatic tails, such as compounds **40** and **41**, still displayed fair binding potential, where the former showed more favorable hydrogen bond interactions than did the latter compound (Figure S3E,F, respectively).

Similarly, a prominent core shared by M1R PAMs and CB2R agonists is the pyrazoloquinolinone scaffold. The primary differences lie in the tail region: M1R-directed analogs possess aromatic tails (compounds **12–20**, Figure 6), whereas CB2R counterparts feature flexible pentyl chains (compounds **42–45**, Figure 8). Docking studies revealed that compounds **42** and **43** engage in key interactions, including aromatic stacking with W400^7.35^ and/or Y179^ECL2^, hydrogen bonding with Q177^ECL2^, and favorable orientation of the pentyl tail within the TMHs 2 and 7 hydrophobic pocket (Figures 9F and S3G, respectively). Conversely, compounds **44** and **45**, which incorporate an extended linker and a bulky adamantanyl head, respectively, display significantly lower interaction quality, likely due to steric hindrance disrupting optimal positioning within the binding site.

The pyrid-4-one carboxamide scaffold was highly represented (compounds **46–56**, Figure 8). Top binders, such as compounds **46–48**, combine cyclohexyl or tetralin heads with a 6-phenyl substitution. These compounds effectively engaged in stacking with W400^7.35^ and/or Y179^ECL2^ while simultaneously forming hydrogen bonds with K392^ECL3^ and/or Q177^ECL2^ without encountering steric hindrance (Figures 9G and S3H,I, respectively). Despite the fact that these derivatives possess monocyclic aromatic cores, the 6-phenyl group seems to compensate by permitting additional stacking. Superposition of these ligand docking poses with those of bi/tricyclic analog docking showed favorable spatial overlaps, suggesting that the phenyl group is well accommodated when oriented orthogonally to the pyridone ring. Nevertheless, their flexible hydrocarbon tails may limit the binding efficacy by favoring van der Waals interactions over robust aromatic stacking. While derivatives featuring adamantanyl or adamantanyl-methyl head groups or branched substituents at position 6 were too bulky and lacked compatibility with the pocket.

Similarly, quinolinone carboxamide derivatives (Compounds **57–60**, Figure 8), featuring a fused bicyclic core this time, were evaluated for binding. Among them, only compound **60**, which incorporates a tetralin headgroup, showed a favorable binding profile, outperforming analogs bearing bulkier headgroups (Figure 9H).

Another scaffold was the indole carboxamide, characterized by a single carboxamide group (Compounds **61–69**, Figure 8), in contrast to the dicarboxamide structure seen in compound **36**. Notably, derivatives bearing a naphthyl or cyclohexyl substituent at the carboxamide group exhibited the best fit within the binding site, striking an intriguing pose reminiscent of the selective M2R PAM LY2119620, as observed in M2R–IXO–PAM complex structures (PDB: 4MQT^25^ and 6OIK^63^). In this orientation, the indole core is preserved for π–π stacking with W400^7.35^ and Y179^ECL2^, while the carboxamide moiety is directed toward the TMHs 2 and 7 region this time (Figures 9I and S3J). Here, the carbonyl oxygen forms a hydrogen bond with Y82^2.60^, and the amide nitrogen is positioned to interact with E397^ECL3^, potentially via a water-mediated hydrogen bond. Additionally, the naphthalene ring in compound **61** further stabilized the binding via aromatic stacking with Y85^2.63^. While other derivatives that possess a bulky adamantanyl headgroup were unable to fit within the binding site and demonstrated limited binding.

A similar binding orientation was observed for the indolyl-phenyl methanone derivatives (Compounds **70–71**, Figure 8). Within this series, compound **70** outperformed compound **71**, likely due to the presence of a hydrophilic *N*-substituent in compound **70** that extended toward ECL3, enabling an extra ion–dipole interaction with K392^ECL3^ (Figures 9J and S3K, respectively). Nonetheless, while these results are encouraging, further validation will be necessary to validate the proposed binding modes for both scaffold classes.

Conversely, several scaffold series demonstrated poor suitability for the M1R PAM site due to insufficient size, overly short structures, or excessive steric bulk. The dicyclohexylpicolinamide derivatives (Compounds **72–73**, Figure 8), featuring monocyclic aromatic cores and short cyclohexyl tails, result in shallow surface-level binding with weak interaction profiles. Similarly, the adamantanyl indolinyl or quinolinyl methanone derivatives (Compounds **74–76**, Figure 8) were unable to form critical contacts due to their diminutive size and the steric impediment created by the bulky adamantanyl group. Their structural resemblance to dopaminergic modulators may also raise concerns about off-target effects. The pyrazole carboxamide scaffold (Compound **77**, Figure 8), represented by a single Y-shaped molecule bearing a bulky trimethylbicycloheptanyl head, was excessively sterically hindered, preventing effective access to the binding pocket and precluding the formation of critical hydrogen bonds. Lastly, pyrid-2-one carboxamide derivatives (Compounds **78–80**, Figure 8) suffered from steric congestion, particularly in the tetrasubstituted analogue, leading to poor accommodation within the M1R PAM site.

## Conclusion

Our multistrategy computational pipeline for MTDL candidate selection establishes a strong basis for addressing the discovery of dual CB2R-M1R modulators. Pharmacological network analysis not only supported the potential relevance of such dual modulators in AD–related pathways but also revealed their broader multitarget potential for application in associated neurological illnesses. Prior studies have demonstrated the synergistic benefits of cotargeting the endocannabinoid and cholinergic systems in AD.^33,80,81^ For example, one research team developed hybrid dual-acting compounds that both inhibit butyrylcholinesterase (BChE) while activating CB2R,^82^ and another pursued a molecule combining acetylcholinesterase (AChE)/BChE inhibition with CB2R modulation.^83^ Both strategies yielded compounds with neuroprotective effects in vitro and demonstrated cognitive improvement in vivo.

Compared with these prior strategies, our approach simultaneously targets two GPCRs—CB2R and M1R—both embedded in the lipid bilayer, offering the advantage that receptors share similar membrane-associated localization, signal transduction mechanisms, and drug-accessibility properties. Such spatial and functional alignment increases the likelihood of achieving comparable pharmacokinetic and pharmacodynamic profiles within a single chemotype, in contrast to the challenges often encountered when dual ligands combine mechanistically unrelated targets, such as receptors and enzymes. Importantly, the design is based on a single chemotype capable of modulating both receptors rather than relying on hybridization or linkage of separate pharmacophores. While previous hybrid ligands, including CB2R–cholinesterase^82,83^ and CB2R–FAAH^67^ duals, have demonstrated therapeutic promise, they often display suboptimal drug-likeness due to their bulky scaffolds, elevated molecular weight, excessive lipophilicity, and limited brain penetration.

Moreover, while cholinesterase inhibitors act indirectly to increase acetylcholine and are frequently associated with peripheral side effects like gastrointestinal and hepatotoxicity,^84^ M1R PAM directly stimulates central cholinergic signaling pathways with enhanced specificity for cognition-related circuits in the CNS. Collectively, this dual GPCR approach has the potential to deliver improved drug-likeness, greater brain penetration, enhanced safety, and a more precise disease-modifying impact in neurodegenerative disorders.

Building upon these insights, our approach can also be viewed as a computational repurposing strategy, in which compounds previously validated for activity on one receptor undergo systematic evaluation and optimization to extend their utility for a secondary, mechanistically related target. This strategy leverages existing knowledge of bioactivity to enhance the reliability of predicted interactions, mitigate safety concerns during subsequent experimental validation, and optimize the utilization of known chemical entities. This approach provides a logical and resource-efficient method for identifying new MTDLs with improved translational feasibility.

PCA of MACCS FBs revealed significant chemical overlaps between CB2R agonists and M1R PAMs, supporting the feasibility of identifying dual-target candidates. The subsequent integration of diverse molecular descriptor profiling and comprehensive docking analysis further enabled the efficient shortlisting of promising dual modulators. Scaffold analysis identified chemotypes with structural features suitable for engaging both binding sites. Notably, several CB2R agonists and M1R PAMs share core structures such as pyrazoloquinolinone (Figure 6: 12–18; Figure 8: 42–43) and indole carboxamide (Figure 6: 1–2; Figure 8: 61–62), highlighting their suitability for future pharmacophore modeling and SAR-guided optimization.

Collectively, these findings underscore the value of integrative computational strategies—combining pharmacological profiling, molecular descriptor analysis, and structure-based modeling—in the rational design of MTDL relevant to neurodegenerative disorders. The generated results offer testable, hypothesis-driven insights that serve as evidence-based computational resources to guide experimental scientists, thereby contributing to bridging the gap between computational discovery and experimental validation. We acknowledge that the present work is entirely computational in nature; therefore, experimental confirmation through in vitro binding assays, functional cellular studies, and in vivo evaluations in AD models will be crucial next steps to verify and refine these predictions. Accordingly, we outline a clear roadmap for future studies aimed at validating and expanding upon these computational findings.

## Supplementary Material





## Data Availability

Cleaned executable scripts and documentation are available at: https://github.com/ihisawi/Custom-Python-scripts-for-generating-Fingerprints.git

## Notes

Fingerprint notations along with the open-source software packages used for their calculation; chemical structures of M1R PAMs selected to investigate their potential cross-affinity with CB2R using IFD; chemical structures of CB2R agonist compounds selected to investigate their potential cross-affinity with M1R using IFD; superimposition of docked and cocrystallized poses of CB2R agonists; key interactions between the M1R PAMs docked within the CB2R orthosteric binding pocket; and key interactions between the CB2R agonists docked within the M1R allosteric binding pocket (PDF)
Cleaned data of CB2R agonists and M1R PAMs (XLSX)

## Abbreviations

CB2R: cannabinoid receptor type 2
M1R: muscarinic acetylcholine receptor subtype 1
MTDLs: multitarget directed ligands
GPCRs: G-protein coupled receptors
AD: Alzheimer’s Disease
PAMs: positive allosteric modulators
FPs: fingerprints
*N*,*N*: nearest neighbor
PPIs: protein–protein interactions
FDRs: false discovery rates
PCA: principal component analysis
Tc: Tanimoto coefficients
IFD: induced fit docking
XP: extra precision
KEGG: Kyoto Encyclopedia of Genes and Genomes
TMHs: transmembrane helices
ECL: Extracellular loop
SAR: structure-activity relationship
IXO: Iperoxo
