# Supplementary material for: Computational Studies toward the Identification of CB2R-M1R Dual Modulators
Source: ACS Omega. 2026 Feb 19;11(8):13121–36. doi: 10.1021/acsomega.5c07866 (PMC12961564; doi:10.1021/acsomega.5c07866)
Supplement: Supplementary file 1 [file ao5c07866_si_001.pdf]

SUPPORTING INFORMATION

Computational Studies Towards the Identification of CB2R-M1R Dual Modulators

Israa H. Isawi[1], Rufaida Al-Zoubi[1], Rima Hajjo[2], Rayan M. Obeidat[1], Islam H. AlKhawaldeh[1], Omar M. Al Kilani[1], Mahmoud J. Alhaj Hasan[3], Paula Morales[4*]*

[1]Department of Medicinal Chemistry and Pharmacognosy, Faculty of Pharmacy, Jordan University of Science and Technology, Irbid, 22110, Jordan

[2]Department of Pharmacy, Faculty of Pharmacy, Al-Zaytoonah University of Jordan, Amman 11733, Jordan

[3]Department of Computer Science, Faculty of Computer and Information Technology, Jordan University of Science and Technology, Irbid, 22110, Jordan

[4]Instituto de Química Médica, Consejo Superior de Investigaciones Científicas (IQM-CSIC), Madrid, 28006, Spain

*Corresponding Authors: ihisawi@just.edu.jo; paula.morales@iqm.csic.es

Table S1. *Fingerprint notations along with the open-source software packages used for their calculation.*

Fingerprints Name	Description	Number of Bits	Package	Reference
**MACCS**#	MACCS fingerprint that generates 166-bit MACCS keys	166	CDK	[1,2]
**PUB**#	PubChem fingerprints that generate 881-bit-long structural key	881	CDK	[3]
**KR***	Klekota-Roth fingerprints based on 4860 substructures	4860	CDK	[4]
**GRAPH***	Graph fingerprint which does not take bond orders into account	1024	CDK	[1,2]
**SUB***	Bit set type fingerprint based on 307 substructures	307	CDK	[1,2]
**HYB***	Fingerprint based on hybridization state of atoms	1024	CDK	[1,2]
**MFP2**#	Morgan connectivity invariants (ECFP-like) with radius = 2	2048	RDKit	[5]
**FeatMFP2**#	Morgan feature invariants (FCFP-like) with radius = 2	2048	RDKit	[5]
**PAIR**#	Atom pairs fingerprints	2048	RDKit	[6]
**PAT**#	SMARTS Pattern fingerprints	2048	RDKit	[5]
**RDKIT**#	Daylight-like fingerprints	2048	RDKit	[5]
**TORS**#	Topological torsion fingerprints	2048	RDKit	[7]
**FP2***	Indexes linear fragments up to 7 atoms		Pybel	[8]

# Fingerprints generated using KNIME fingerprint nodes

*** Fingerprints generated using custom Python scripts**

Table S2. *Chemical Structures of M1R PAMs Selected to Investigate their Potential Cross-Affinity with CB2R using IFD*.

ChEMBL ID	Chemical Structure	IP/EC_{50} (nM) Or Activity	SMILES	Reference
**(1)** **CHEMBL4224929**	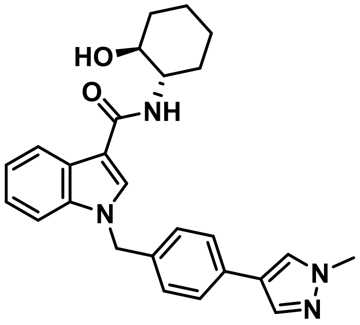	active	Cn1cc(-c2ccc(Cn3cc(C(=O)N[C@H]4CCCC[C@@H]4O)c4ccccc43)cc2)cn1	9
**(2)** **CHEMBL4227499**	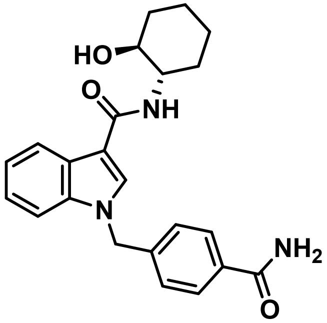	active	NC(=O)c1ccc(Cn2cc(C(=O)N[C@H]3CCCC[C@@H]3O)c3ccccc32)cc1	9

**(3)**<br>**CHEMBL4227561**	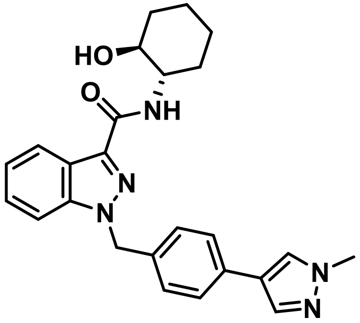	active	Cn1cc(-c2ccc(Cn3nc(C(=O)N[C@H]4CCCC[C@@H]4O)c4ccccc43)cc2)cn1	9
**(4)**<br>**CHEMBL3745855**	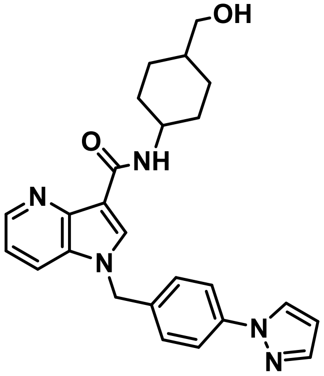	EC_{50}= 248	O=C(N[C@H]1CC[C@H](CO)CC1)c1cn(Cc2ccc(-n3cccn3)cc2)c2cccnc12	10
**(5)**<br>**CHEMBL3745995**	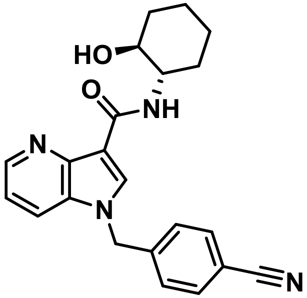	EC_{50}=478	N#Cc1ccc(Cn2cc(C(=O)N[C@H]3CCCC[C@@H]3O)c3ncccc32)cc1	10

**(6)**<br>**CHEMBL3746904**	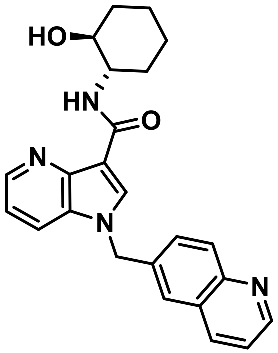	EC_{50}=185	O=C(N[C@H]1CCCC<br>[C@@H]1O)c1cn(Cc2<br>ccc3ncccc3c2)c2cccnc<br>12	10
**(7)**<br>**CHEMBL4759834**	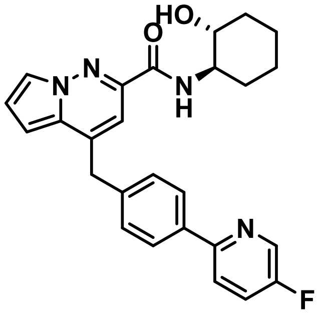	EC_{50}=149	O=C(N[C@@H]1CCC<br>C[C@H]1O)c1cc(Cc2c<br>cc(c3ccc(F)cn3)cc2)c2<br>cccn2n1	11
**(8)**<br>**CHEMBL4783540**	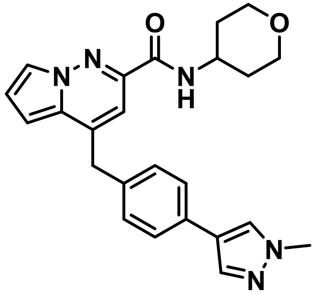	EC_{50}=182	Cn1cc(c2ccc(Cc3cc(C(<br>=O)NC4CCOCC4)nn<br>4cccc34)cc2)cn1	11

**(9)** **CHEMBL474500l**	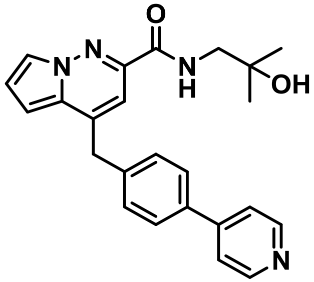	EC_{50}=405	CC(C)(O)CNC(=O)c1cc(Cc2ccc(c3ccncc3)cc2)c2cccn2n1	11
**(10)** **(CID 53307538)**	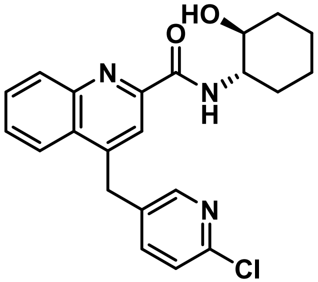	EC_{50}=63	O=C(N[C@H]1CCCC[C@@H]1O)c1cc(Cc2ccc(Cl)nc2)c2ccccc2n1	12
**(11)** **(CID 53307539)**	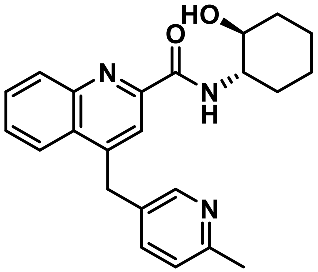	EC_{50}=102	Cc1ccc(Cc2cc(C(=O)N[C@H]3CCCC[C@@H]3O)nc3ccccc23)cn1	12

**(12)**<br>**CHEMBL3360928**	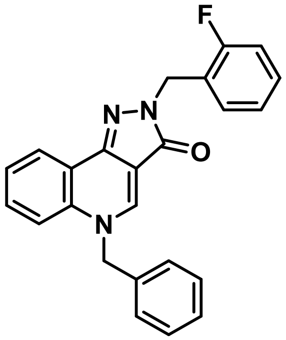	EC_{50}=1000	O=c1c2cn(Cc3ccccc3)<br>c3ccccc3c-<br>2nn1Cc1ccccc1F	13
**(13)**<br>**CHEMBL3360931**	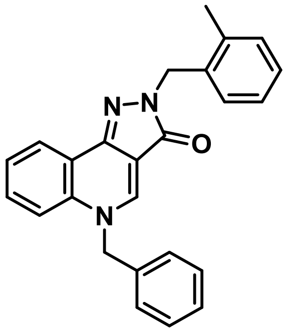	EC_{50}=776.25	Cc1ccccc1Cn1nc2c3cc<br>ccc3n(Cc3ccccc3)cc-<br>2c1=O	13
**(14)**<br>**CHEMBL3360938**	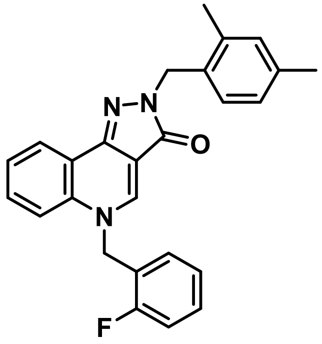	EC_{50}=656	Cc1ccc(Cn2nc3c4cccc<br>c4n(Cc4ccccc4F)cc-<br>3c2=O)c(C)c1	13

**(15)**<br>**CHEMBL3360947**	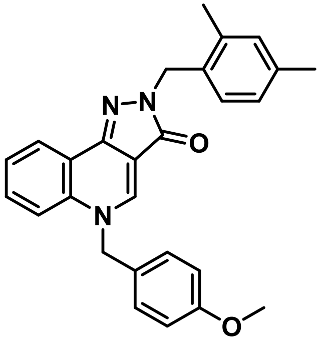	EC_{50}=812.83	COc1ccc(Cn2cc3c(=O)n(Cc4ccc(C)cc4C)nc-3c3ccccc32)cc1	13
**(16)**<br>**CHEMBL4218803**	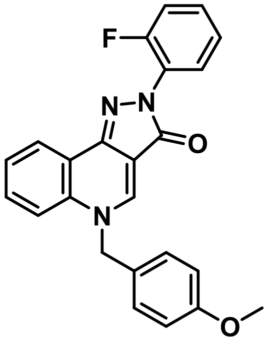	EC_{50}=410	COc1ccc(Cn2cc3c(=O)n(-c4ccccc4F)nc-3c3ccccc32)cc1	14
**(17)**<br>**CHEMBL1556989**	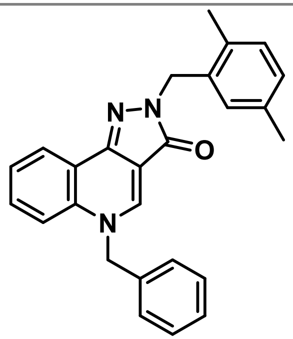	EC_{50}=473	Cc1ccc(C)c(Cn2nc3c4ccccc4n(Cc4ccccc4)cc-3c2=O)c1	13

**(18)** **CHEMBL3360959**	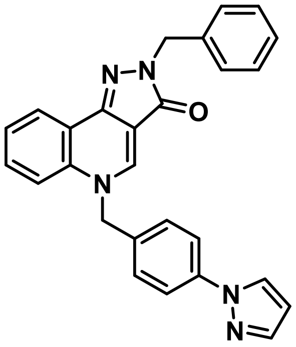	EC_{50}>10	O=c1c2cn(Cc3ccc(-n4cccn4)cc3)c3ccccc3c-2nn1Cc1ccccc1	13
**(19)** **CHEMBL3360954**	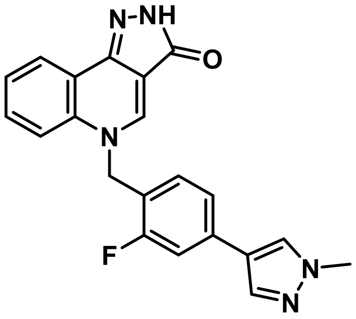	EC_{50}=0.6	Cn1cc(-c2ccc(Cn3cc4c(=O)[nH]nc4c4ccccc43)c(F)c2)cn1	13
**(20)** **CHEMBL3360955**	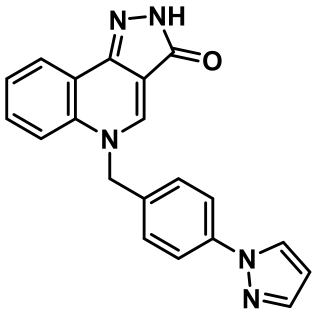	EC_{50}=1.2	O=c1[nH]nc2c3ccccc3n(Cc3ccc(n4cccn4)cc3)cc1-2	13

**(21)** **CHEMBL2203584**	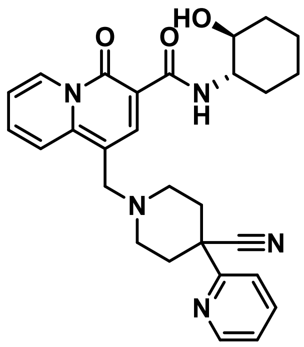	IP=381	CO[C@H]1CCCC[C@@H]1NC(=O)c1cc(CN2CCC(C#N)(c3ccccn3)CC2)c2ccccn2c1=O	15
**(22)** **CHEMBL2204291***	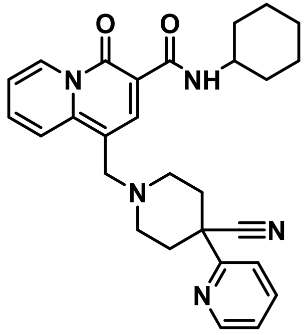	IP=253	N#CC1(c2ccccn2)CCN(Cc2cc(C(=O)NC3CCCCC3)c(=O)n3ccccc23)CC1	15
**(23)** **CHEMBL2204292**	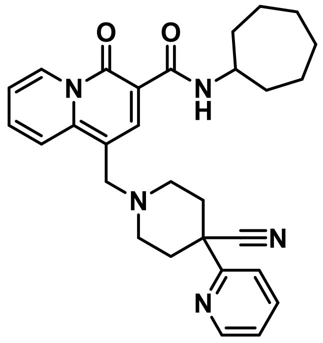	IP=304	N#CC1(c2ccccn2)CCN(Cc2cc(C(=O)NC3CCCCCC3)c(=O)n3ccccc23)CC1	15

**(24)** **CHEMBL2204294***	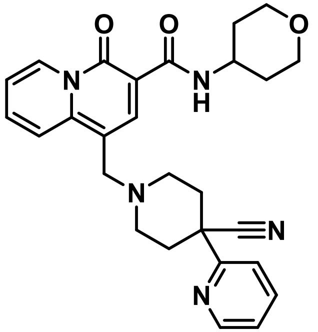	IP=338	N#CC1(c2ccccn2)CCN(Cc2cc(C(=O)NC3CCOCC3)c(=O)n3ccccc23)CC1	15
**(25)** **CHEMBL2204297**	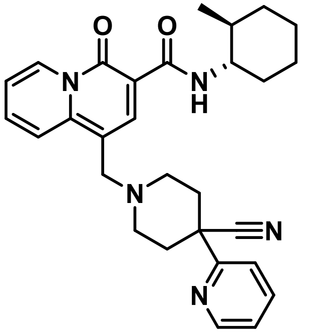	IP=190	C[C@H]1CCCC[C@@H]1NC(=O)c1cc(CN2CCC(C#N)(c3ccccn3)CC2)c2ccccn2c1=O	15
**(26)** **CHEMBL2204298**	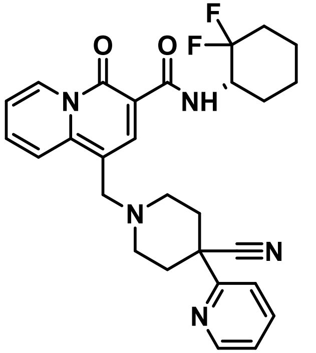	IP=205	N#CC1(c2ccccn2)CCN(Cc2cc(C(=O)NC3CCCCC3(F)F)c(=O)n3ccccc23)CC1	15

**(27)** **CHEMBL1090104**	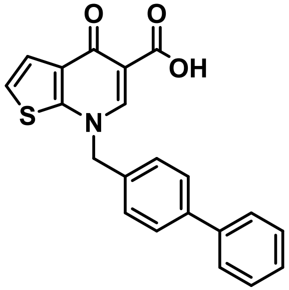	IP=370	O=C(O)c1cn(Cc2ccc(-c3ccccc3)cc2)c2sccc2c1=O	16
**(28)** **CHEMBL3360956**	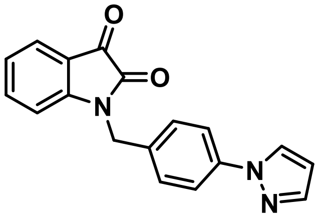	EC_{50}=10	O=C1C(=O)N(Cc2ccc(n3cccn3)cc2)c2ccccc21	13

*** Compounds have affinity on P-glycoprotein (P-gp) efflux transporters**

Table S3. *Chemical Structures of CB2R Agonist Compounds Selected to Investigate their Potential Cross-Affinity with M1R using IFD.*

ChEMBL ID	Chemical Structure	EC_{50} (nM)	SMILES	Reference
**(29)**<br>**CHEMBL456535**	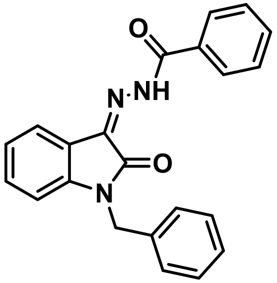	63.7	O=C(N/N=C1\C(=O)<br>N(Cc2ccccc2)c2ccccc2<br>1)c1ccccc1	[17]
**(30)**<br>**CHEMBL3354953**	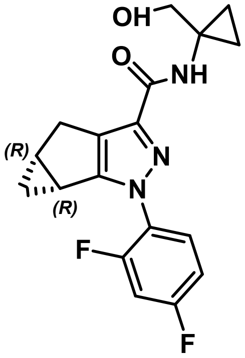	13.6	O=C(NC1(CO)CC1)c<br>1nn(-<br>c2ccc(F)cc2F)c2c1C[C<br>@H]1C[C@@H]21	[18]

**(30′)** **CHEMBL3354964**	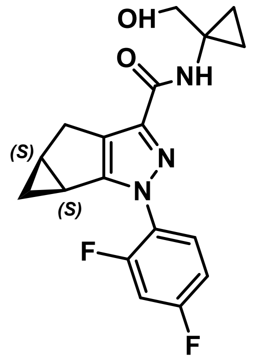	24.8	O=C(NC1(CO)CC1)c1nn(-c2ccc(F)cc2F)c2c1C[C@@H]1C[C@H]21	18
**(31)** **CHEMBL3354954**	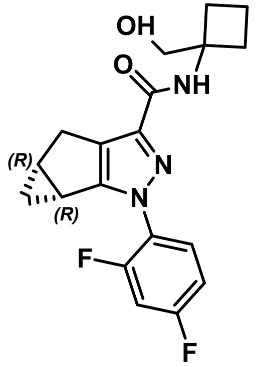	1.2	O=C(NC1(CO)CCC1)c1nn(-c2ccc(F)cc2F)c2c1C[C@H]1C[C@@H]21	18
**(32)** **CHEMBL3354955**	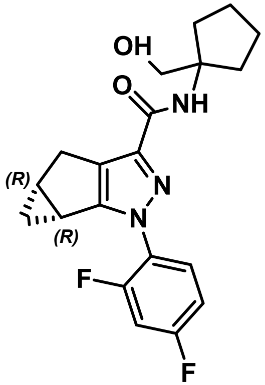	0.7	O=C(NC1(CO)CCCC1)c1nn(-c2ccc(F)cc2F)c2c1C[C@H]1C[C@@H]21	18

**(33)**<br>**CHEMBL3354957**	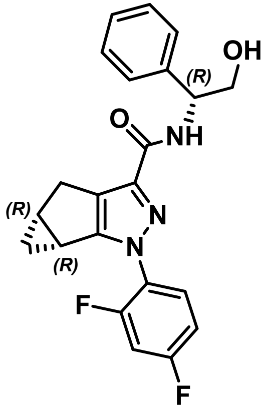	5.4	O=C(N[C@@H](CO)c<br>1ccccc1)c1nn(-<br>c2ccc(F)cc2F)c2c1C[C<br>@H]1C[C@@H]21	18
**(33′)**<br>**CHEMBL3354956**	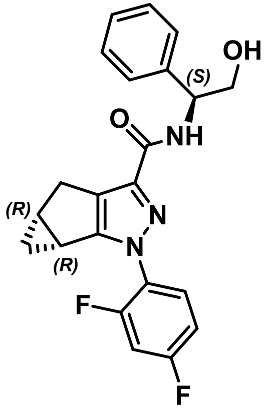	38.8	O=C(N[C@H](CO)c1<br>ccccc1)c1nn(-<br>c2ccc(F)cc2F)c2c1C[C<br>@H]1C[C@@H]21	18
**(34)**<br>**CHEMBL3354967**	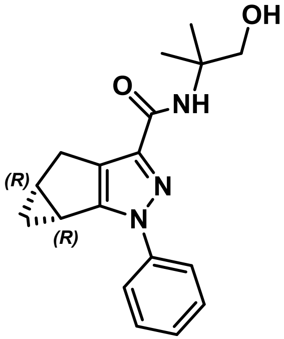	100.9	CC(C)(CO)NC(=O)c1<br>nn(-<br>c2ccccc2)c2c1C[C@H]<br>1C[C@@H]21	18

**(35)**<br>**CHEMBL3354958**	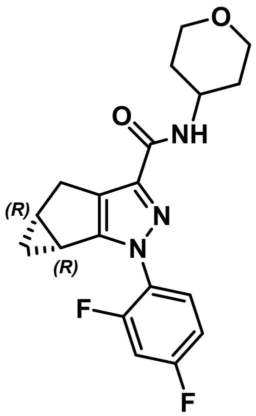	15.2	O=C(NC1CCOCC1)c<br>1nn(-<br>c2ccc(F)cc2F)c2c1C[C<br>@H]1C[C@@H]21	18
**(35′)**<br>**CHEMBL3354965**	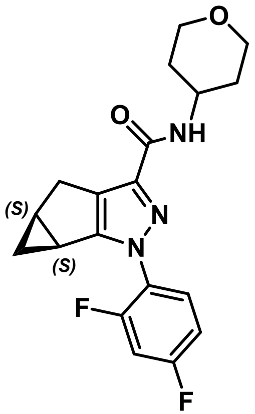	38.5	O=C(NC1CCOCC1)c<br>1nn(-<br>c2ccc(F)cc2F)c2c1C[C<br>@@H]1C[C@H]21	18

**(36)**<br>**CHEMBL5199876**	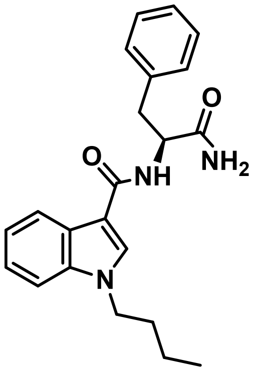	40	CCCCn1cc(C(=O)N[C@@H](Cc2ccccc2)C(N)=O)c2ccccc21	19
**(37)**<br>**CHEMBL5209051**	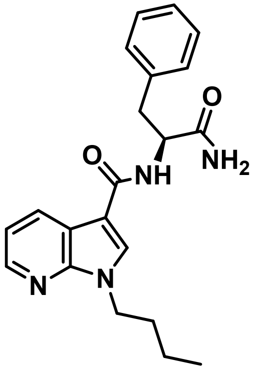	316.23	CCCCn1cc(C(=O)N[C@@H](Cc2ccccc2)C(N)=O)c2cccnc21	19
**(38)**<br>**CHEMBL5178204**	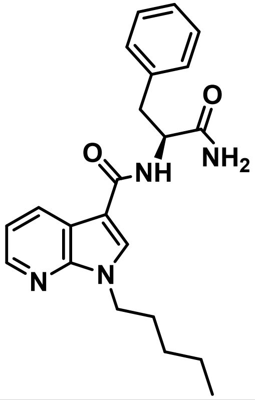	381	CCCCCn1cc(C(=O)N[C@@H](Cc2ccccc2)C(N)=O)c2cccnc21	19

**(39)** **CHEMBL3422790**	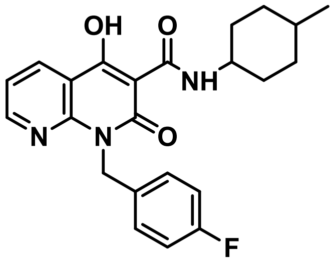	3.162	CC1CCC(NC(=O)c2c(O)c3cccnc3n(Cc3ccc(F)cc3)c2=O)CC1	20
**(40)** **CHEMBL466223**	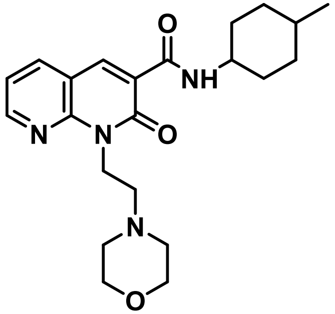	28	CC1CCC(NC(=O)c2cc3cccnc3n(CCN3CCOCC3)c2=O)CC1	21
**(41)** **CHEMBL3422788**	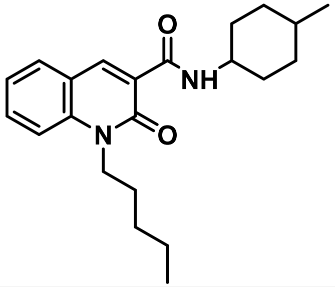	1.995	CCCCCn1c(=O)c(C(=O)NC2CCC(C)CC2)cc2ccccc21	20

**(42)**<br>**CHEMBL3421689**	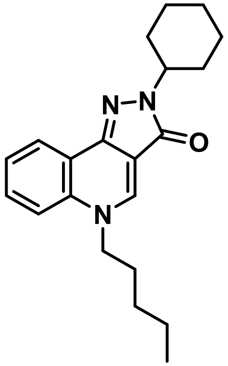	204	CCCCCn1cc2c(=O)n(<br>C3CCCCC3)nc-<br>2c2ccccc21	22
**(43)**<br>**CHEMBL 3421697**	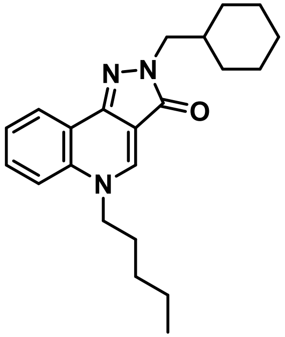	14.3	CCCCCn1cc2c(=O)n(<br>CC3CCCCC3)nc-<br>2c2ccccc21	22
**(44)**<br>**CHEMBL3421698**	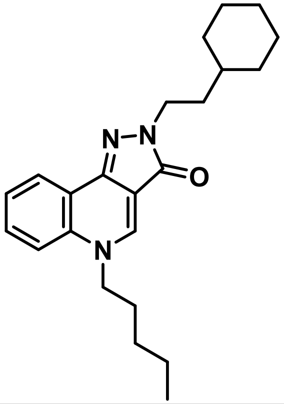	5.4	CCCCCn1cc2c(=O)n(<br>CCC3CCCCC3)nc-<br>2c2ccccc21	22

**(45)** **CHEMBL3421699**	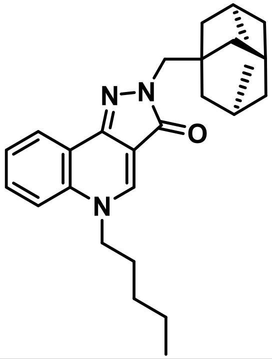	64	CCCCCn1cc2c(=O)n(CC34CC5CC(CC(C5)C3)C4)nc-2c2ccccc21	22
**(46)** **CHEMBL1641947**	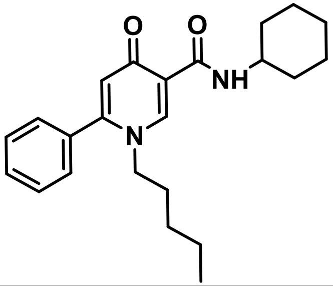	1.9	CCCCCn1cc(C(=O)NC2CCCCC2)c(=O)cc1-c1ccccc1	23
**(47)** **CHEMBL1641948**	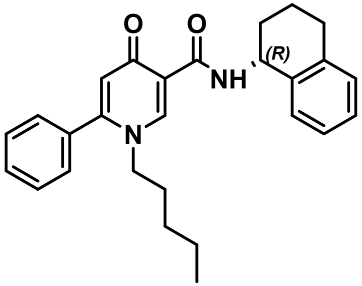	1.9	CCCCCn1cc(C(=O)N[C@@H]2CCCc3ccccc32)c(=O)cc1-c1ccccc1	23

**(48)**<br>**CHEMBL1641949**	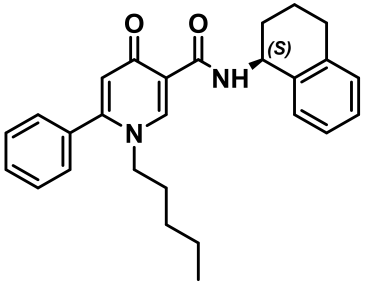	60	CCCCCn1cc(C(=O)N[C@H]2CCCc3ccccc32)c(=O)cc1-c1ccccc1	23
**(49)**<br>**CHEMBL2170545**	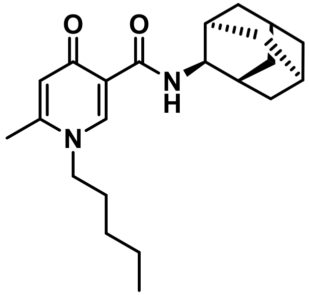	38.4	CCCCCn1cc(C(=O)N[C@H]2[C@@H]3C[C@H]4C[C@@H](C3)C[C@@H]2C4)c(=O)cc1C	24
**(50)**<br>**CHEMBL2170529***	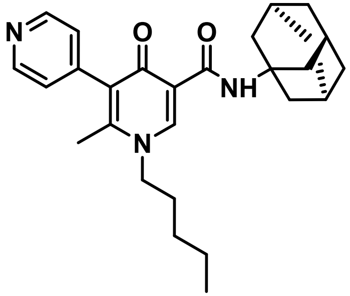	114	CCCCCn1cc(C(=O)N[C@]23C[C@H]4C[C@H](C[C@H](C4)C2)C3)c(=O)c(-c2ccncc2)c1C	24

**(51)** **CHEMBL2170549**	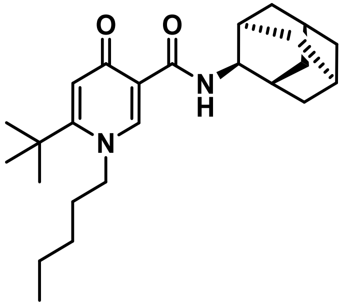	452	CCCCCn1cc(C(=O)N[C@H]2[C@@H]3C[C@H]4C[C@@H](C3)C[C@@H]2C4)c(=O)cc1C(C)(C)C	24
**(52)** **CHEMBL2170544**	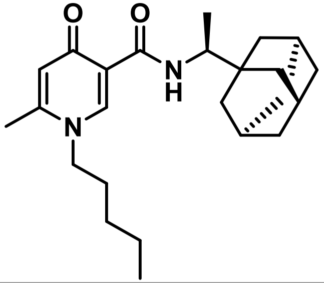	84.2	CCCCCn1cc(C(=O)N[C@@H](C)[C@]23C[C@H]4C[C@H](C[C@H](C4)C2)C3)c(=O)cc1C	24
**(53)** **CHEMBL1642940**	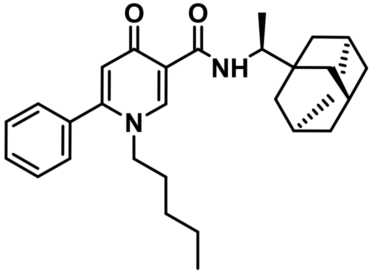	6.5	CCCCCn1cc(C(=O)N[C@@H](C)[C@]23C[C@H]4C[C@H](C[C@H](C4)C2)C3)c(=O)cc1-c1ccccc1	23
**(54)** **CHEMBL1641942**	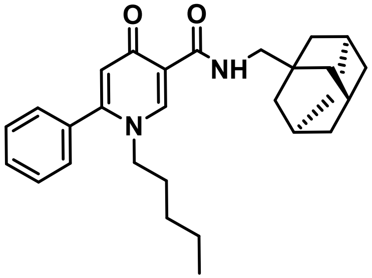	10.9	CCCCCn1cc(C(=O)NC[C@]23C[C@H]4C[C@H](C[C@H](C4)C2)C3)c(=O)cc1-c1ccccc1	23

**(55)**<br>**CHEMBL2170546**	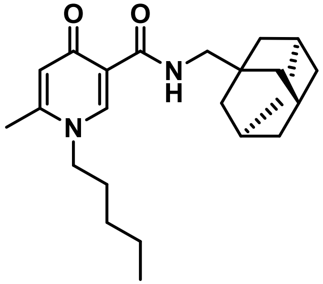	53.1	CCCCCn1cc(C(=O)N<br>C[C@]23C[C@H]4C[<br>C@H](C[C@H](C4)C2<br>)C3)c(=O)cc1C	24
**(56)**<br>**CHEMBL16414938**	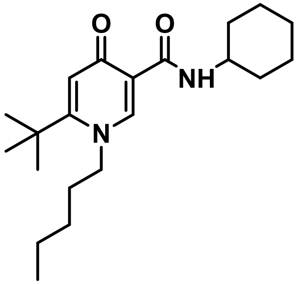	7.8	**CCCCCn1cc(C(=O)N<br>C2CCCCC2)c(=O)cc1<br>C(C)(C)C**	23
**(57)**<br>**CHEMBL2152800**	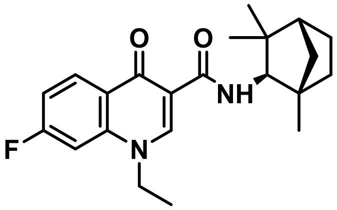	17.2	CCn1cc(C(=O)N[C@<br>@H]2C(C)(C)[C@H]3<br>CC[C@]2(C)C3)c(=O)<br>c2ccc(F)cc21	25
**(58)**<br>**CHEMBL264521**	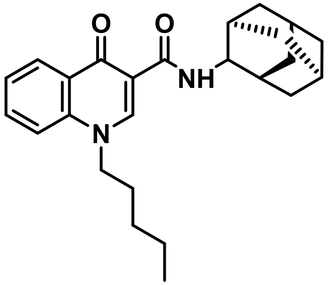	24.8	CCCCCn1cc(C(=O)N<br>[C@H]2[C@@H]3C[C<br>@H]4C[C@@H](C3)C[<br>C@@H]2C4)c(=O)c2cc<br>ccc21	26

**(59)**<br>**CHEMBL372544**	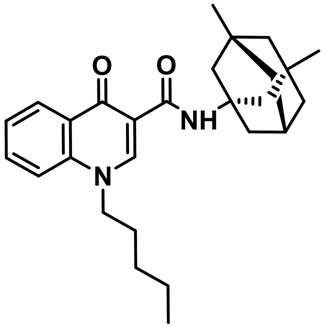	14.1	CCCCCn1cc(C(=O)N[C@@]23C[C@@H]4C[C@@](C)(C[C@@](C)(C4)C2)C3)c(=O)c2ccccc21	26
**(60)**<br>**CHEMBL380610**	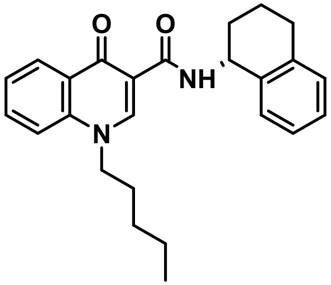	157.4	CCCCCn1cc(C(=O)N[C@H]2CCCc3ccccc32)c(=O)c2ccccc21	26
**(61)**<br>**CHEMBL4079817**	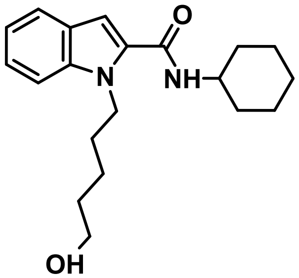	420	O=C(NC1CCCCC1)c1cc2ccccc2n1CCCCCO	27
**(62)**<br>**CHEMBL1828807**	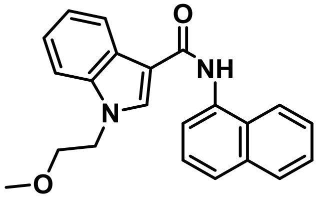	6.31	COCCn1cc(C(=O)Nc2cccc3ccccc23)c2ccccc21	28

**(63)**<br>**CHEMBL4073323**	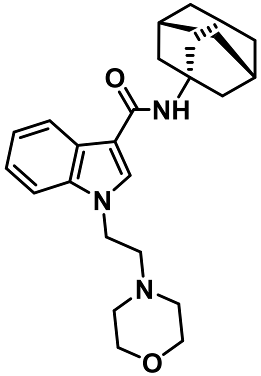	59	O=C(N[C@]12C[C@H]3C[C@H](C[C@H](C3)C1)C2)c1cn(CCN2CCOCC2)c2ccccc12	27
**(64)**<br>**CHEMBL4062303**	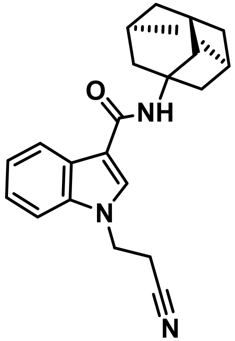	180	N#CCCn1cc(C(=O)N[C@]23C[C@H]4C[C@H](C[C@H](C4)C2)C3)c2ccccc21	27
**(65)**<br>**CHEMBL4087526**	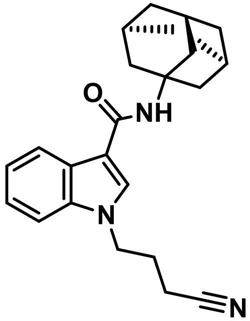	31	N#CCCCn1cc(C(=O)N[C@]23C[C@H]4C[C@H](C[C@H](C4)C2)C3)c2ccccc21	27

**(66)**<br>**CHEMBL4090311**	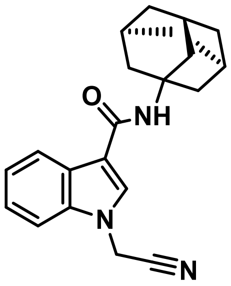	410	N#CCn1cc(C(=O)N[C@]23C[C@H]4C[C@H](C[C@H](C4)C2)C3)c2ccccc21	27
**(67)**<br>**CHEMBL4066155**	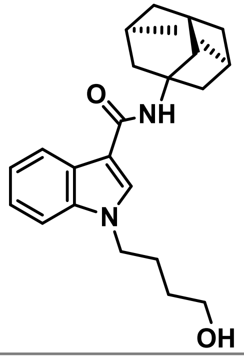	160	O=C(N[C@]12C[C@H]3C[C@H](C[C@H](C3)C1)C2)c1cn(CCCCO)c2ccccc12	27
**(68)**<br>**CHEMBL4079650**	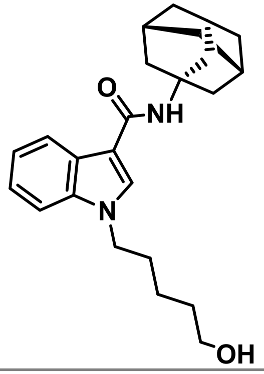	62	O=C(N[C@]12C[C@H]3C[C@H](C[C@H](C3)C1)C2)c1cn(CCCCCO)c2ccccc12	27

**(69)**<br>**CHEMBL4103902**	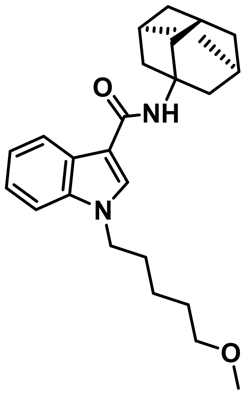	82	COCCCCCn1cc(C(=O)N[C@]23C[C@H]4C[C@H](C[C@H](C4)C2)C3)c2ccccc21	27
**(70)**<br>**CHEMBL4284943**	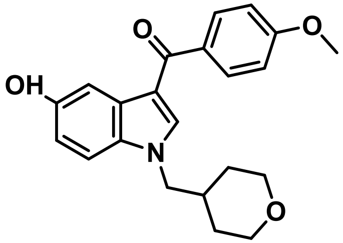	4.4	COc1ccc(C(=O)c2cn(CC3CCOCC3)c3ccc(O)cc23)cc1	29
**(71)**<br>**CHEMBL4089341**	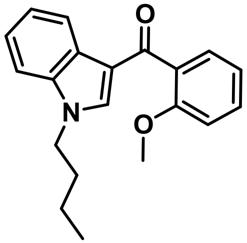	4.5	CCCCn1cc(C(=O)c2ccccc2OC)c2ccccc21	30

**(72)**<br>**CHEMBL3934189**	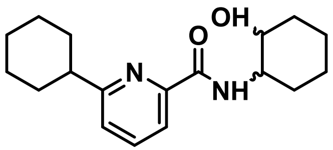	291.8	O=C(N[C@H]1CCCC[C@@H]1O)c1cccc(C2CCCCC2)n1	31
**(73)**<br>**CHEMBL3928538**	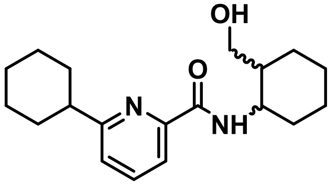	157.6	O=C(N[C@@H]1CCCC[C@H]1CO)c1cccc(C2CCCCC2)n1	31
**(74)**<br>**CHEMBL2338178**	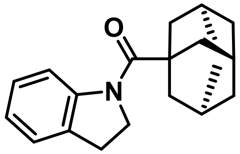	23	O=C(N1CCc2ccccc21)[C@]12C[C@H]3C[C@H](C[C@H](C3)C1)C2	32
**(75)**<br>**CHEMBL2338181**	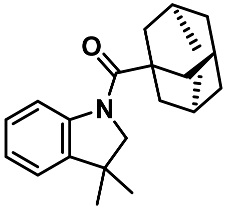	16	CC1(C)CN(C(=O)[C@]23C[C@H]4C[C@H](C[C@H](C4)C2)C3)c2ccccc21	32

**(76) CHEMBL246952**	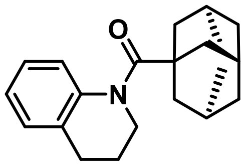	72	O=C(N1CCCc2ccccc2 1)[C@]12C[C@H]3C[C@H](C[C@H](C3)C1)C2	32
**(77) CHEMBL381689**	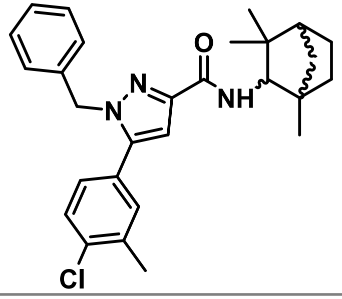	1.8	Cc1ccc(Cn2nc(C(=O) N[C@H]3C(C)(C)[C@ @H]4CC[C@@]3(C)C 4)cc2- c2ccc(Cl)c(C)c2)cc1	23
**(78) CHEMBL2022698**	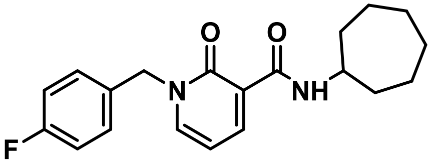	6.31	O=C(NC1CCCCCC1) c1cccn(Cc2ccc(F)cc2)c 1=O	33
**(79) CHEMBL4175577**	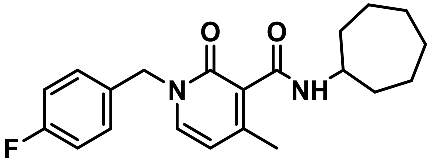	1.9	Cc1ccn(Cc2ccc(F)cc2) c(=O)c1C(=O)NC1CC CCCC1	34

**(80) CHEMBL4167303**	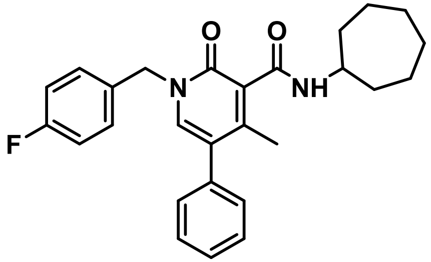	12	Cc1c(-c2ccccc2)cn(Cc2ccc(F)cc2)c(=O)c1C(=O)NC1CCCCCC1	34

***Compounds have affinity on P-glycoprotein (P-gp) efflux transporters**

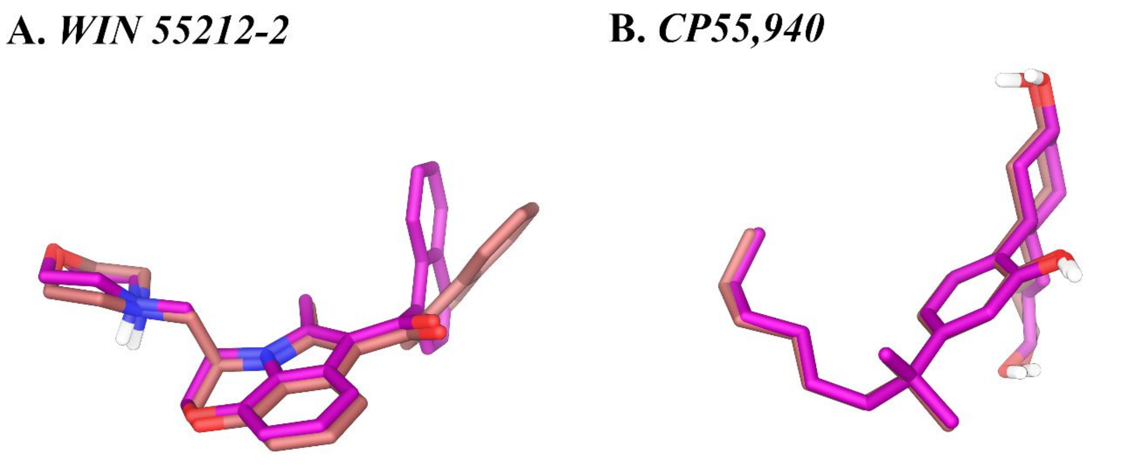

Figure S1. Superimposition of Docked and Co-Crystallized Poses of CB2R Agonists. A. IFD pose of WIN 55,212-2 (magenta) overlaid with its co-crystallized pose (dark rose) from PDB ID: 6PT0, with an RMSD of 1.030 Å. **B.** IFD pose of CP 55,940 (magenta) overlaid with its co-crystallized pose (dark rose) from PDB ID: 8GUR, with an RMSD of 0.402 Å.

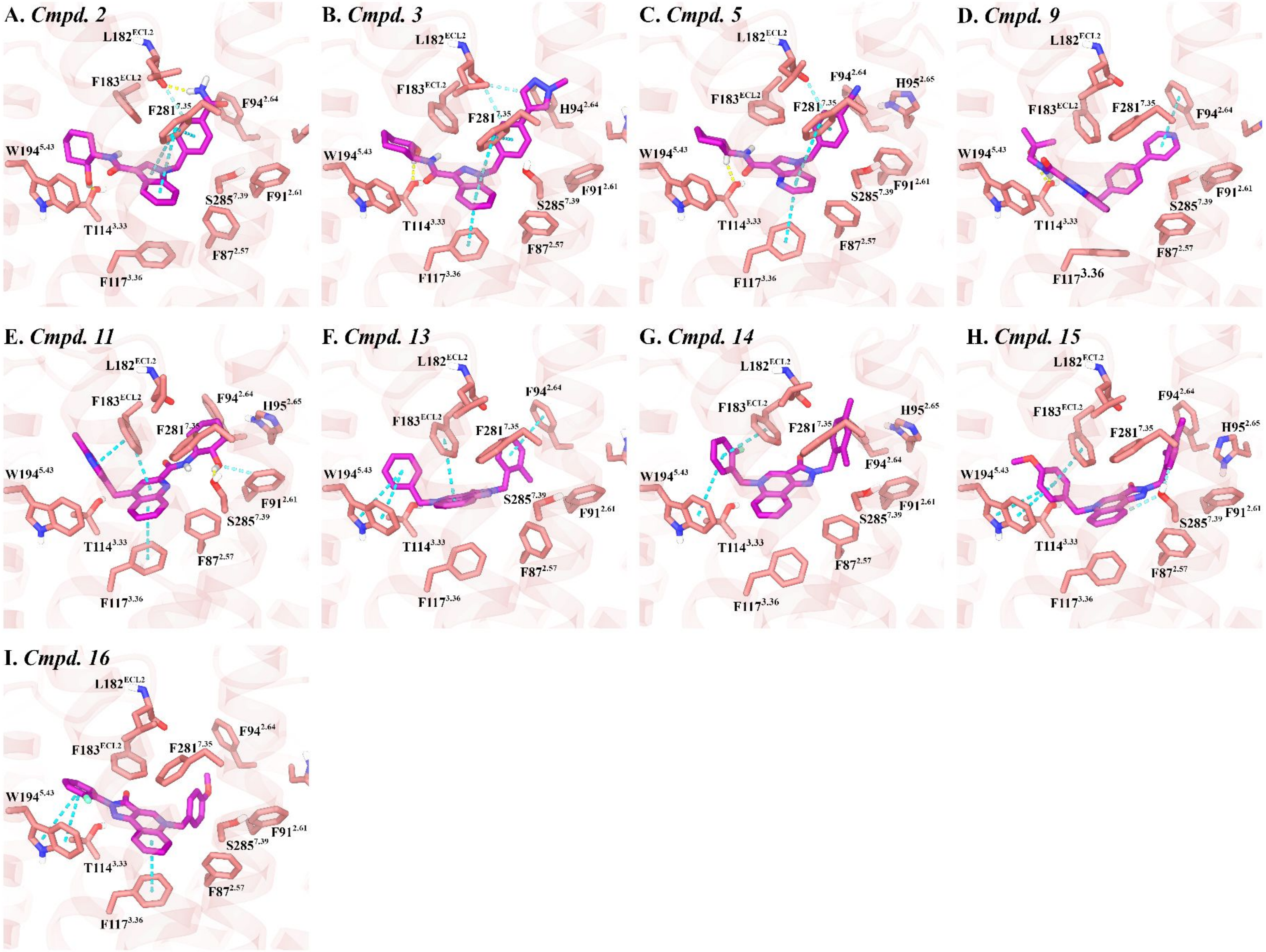
A. Cmpd. 2
B. Cmpd. 3
C. Cmpd. 5
D. Cmpd. 9
E. Cmpd. 11
F. Cmpd. 13
G. Cmpd. 14
H. Cmpd. 15
I. Cmpd. 16

Figure S2. Key Interactions Between the M1R PAMs Docked Within the CB2R Orthosteric Binding Pocket. Key interacting residues for **A.** Compound 2, **B.** Compound 3, **C.** Compound 5, **D.** Compound 9, **E.** Compound 11, **F.** Compound 13, **G.** Compound 14, **H.** Compound 15, and **I.** Compound 16, all docked into the orthosteric site of CB2R (PDB ID: 8GUR or 6PT0). CB2R is shown as rose ribbons and key ligand-binding residues are displayed as dark rose sticks. Ligands are represented as magenta sticks within their respective binding sites. Hydrogen bonds are indicated with yellow dashed lines, π–π stacking interactions with cyan dashed lines, aromatic hydrogen bonds with light cyan dashed lines, and π–cation interactions with green dashed lines.

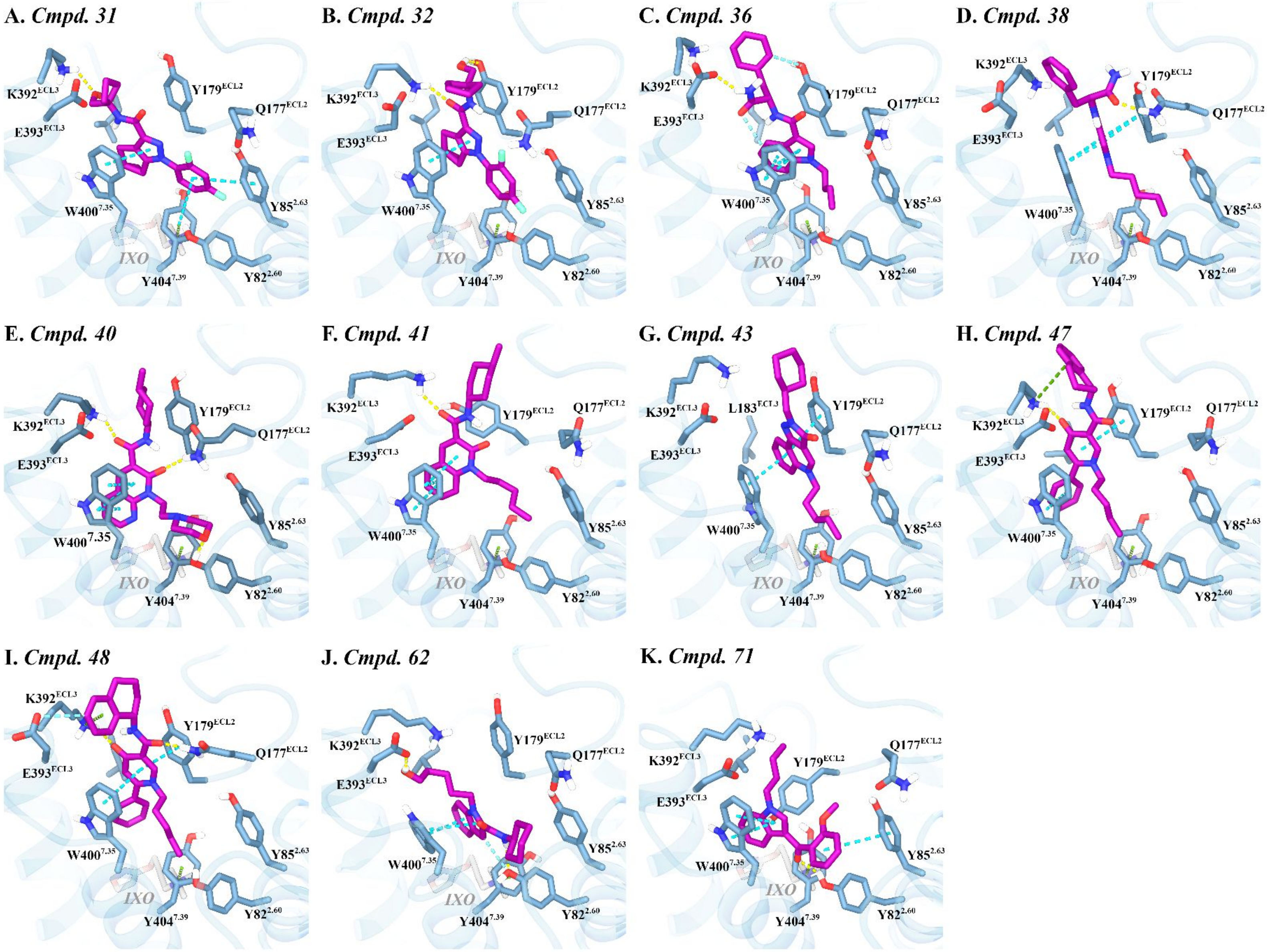
A. Cmpd. 31
K392^{ECL3}
E393^{ECL3}
Y179^{ECL2}
Q177^{ECL2}
W400$^{7.35}$
Y85$^{2.63}$
IXO
Y404$^{7.39}$
Y82$^{2.60}$
B. Cmpd. 32
K392^{ECL3}
E393^{ECL3}
Y179^{ECL2}
Q177^{ECL2}
W400$^{7.35}$
Y85$^{2.63}$
IXO
Y404$^{7.39}$
Y82$^{2.60}$
C. Cmpd. 36
K392^{ECL3}
E393^{ECL3}
Y179^{ECL2}
Q177^{ECL2}
W400$^{7.35}$
Y85$^{2.63}$
IXO
Y404$^{7.39}$
Y82$^{2.60}$
D. Cmpd. 38
K392^{ECL3}
Y179^{ECL2}
E393^{ECL3}
Q177^{ECL2}
W400$^{7.35}$
Y85$^{2.63}$
IXO
Y404$^{7.39}$
Y82$^{2.60}$
E. Cmpd. 40
K392^{ECL3}
Y179^{ECL2}
E393^{ECL3}
Q177^{ECL2}
W400$^{7.35}$
Y85$^{2.63}$
IXO
Y404$^{7.39}$
Y82$^{2.60}$
F. Cmpd. 41
K392^{ECL3}
Y179^{ECL2}
Q177^{ECL2}
E393^{ECL3}
W400$^{7.35}$
Y85$^{2.63}$
IXO
Y404$^{7.39}$
Y82$^{2.60}$
G. Cmpd. 43
K392^{ECL3}
L183^{ECL3}
Y179^{ECL2}
Q177^{ECL2}
E393^{ECL3}
W400$^{7.35}$
Y85$^{2.63}$
IXO
Y404$^{7.39}$
Y82$^{2.60}$
H. Cmpd. 47
K392^{ECL3}
Y179^{ECL2}
Q177^{ECL2}
E393^{ECL3}
W400$^{7.35}$
Y85$^{2.63}$
IXO
Y404$^{7.39}$
Y82$^{2.60}$
I. Cmpd. 48
K392^{ECL3}
Y179^{ECL2}
Q177^{ECL2}
E393^{ECL3}
W400$^{7.35}$
Y85$^{2.63}$
IXO
Y404$^{7.39}$
Y82$^{2.60}$
J. Cmpd. 62
Y179^{ECL2}
K392^{ECL3}
Q177^{ECL2}
E393^{ECL3}
W400$^{7.35}$
Y85$^{2.63}$
IXO
Y404$^{7.39}$
Y82$^{2.60}$
K. Cmpd. 71
Q177^{ECL2}
K392^{ECL3}
Y179^{ECL2}
E393^{ECL3}
W400$^{7.35}$
Y85$^{2.63}$
IXO
Y404$^{7.39}$
Y82$^{2.60}$

Figure S3. Key Interactions Between the CB2R Agonists Docked Within the M1R Allosteric Binding Pocket. Key interacting residues for **A.** Compound 31, **B.** Compound 32, **C.** Compound 36, **D.** Compound 38, **E.** Compound 40, **F.** Compound 41, **G.** Compound 43, **H.** Compound 47, **I.** Compound 48, **J.** Compound 62, and **K.** Compound 71, all docked into the allosteric site of M1R in the presence of the orthosteric agonist Iperoxo (IXO) (PDB ID: 6OIJ). M1R is shown as sky-blue ribbons and key ligand-binding residues are displayed as dark sky-vlue sticks. Ligands are represented as magenta sticks within their respective binding sites. Hydrogen bonds are indicated with yellow dashed lines, π–π stacking interactions with cyan dashed lines, aromatic hydrogen bonds with light cyan dashed lines, and π–cation interactions with green dashed lines.

References

(1) Steinbeck, C.; Han, Y.; Kuhn, S.; Horlacher, O.; Luttmann, E.; Willighagen, E. The chemistry development kit (CDK): an open-source Java library for chemo- and bioinformatics. *J. Chem. Inf. Comput. Sci.* **2003**, *43* (2), 493–500. https://doi.org/10.1021/ci025584y.

(2) Steinbeck, C.; Hoppe, C.; Kuhn, S.; Floris, M.; Guha, R.; Willighagen, E. Recent developments of the chemistry development kit (CDK) – an open-source Java library for chemo- and bioinformatics. *Curr. Pharm. Des.* **2006**, *12* (17), 2111–2120. https://doi.org/10.2174/138161206777585274.

(3) PubChem Substructure Fingerprint. National Center for Biotechnology Information (NCBI), PubChem Project: Bethesda, MD, 2009. https://ftp.ncbi.nlm.nih.gov/pubchem/specifications/pubchem_fingerprints.pdf (accessed September 21, 2024).

(4) Klekota, J.; Roth, F. P. Chemical substructures that enrich for biological activity. *Bioinformatics* **2008**, *24* (21), 2518–2525. https://doi.org/10.1093/bioinformatics/btn479.

(5) Landrum, G. RDKit: Open-source cheminformatics. https://www.rdkit.org/ (accessed September 21, 2024).

(6) Carhart, R. E.; Smith, D. H.; Venkataraghavan, R. Atom pairs as molecular features in structure-activity studies: definition and applications. *J. Chem. Inf. Comput. Sci.* **1985**, *25* (2), 64–73. https://doi.org/10.1021/ci00046a002.

(7) Nilakantan, R.; Bauman, N.; Dixon, J. S.; Venkataraghavan, R. Topological torsion: a new molecular descriptor for SAR applications. Comparison with other descriptors. *J. Chem. Inf. Comput. Sci.* **1987**, *27* (2), 82–85. https://doi.org/10.1021/ci00054a008.

(8) O'Boyle, N. M.; Morley, C.; Hutchison, G. R. Pybel: a Python wrapper for the Open Babel cheminformatics toolkit. *Chem. Cent. J.* **2008**, *2* (1), 5. https://doi.org/10.1186/1752-153X-2-5.

(9) Dallagnol, J. C. C.; Khajehali, E.; van der Westhuizen, E. T.; Jörg, M.; Valant, C.; Gonçalves, A. G.; Capuano, B.; Christopoulos, A.; Scammells, P. J. Synthesis and pharmacological evaluation of heterocyclic carboxamides: positive allosteric modulators of the M1 muscarinic acetylcholine receptor with weak agonist activity and diverse modulatory profiles. *J. Med. Chem.* **2018**, *61* (7), 2875–2894. https://doi.org/10.1021/acs.jmedchem.7b01812.

(10) Davoren, J. E.; O'Neil, S. V.; Anderson, D. P.; Brodney, M. A.; Chenard, L.; Dlugolenski, K.; Edgerton, J. R.; Green, M.; Garnsey, M.; Grimwood, S.; Harris, A. R.; Kauffman, G. W.; Lachapelle, E.; Lazzaro, J. T.; Lee, C. W.; Lotarski, S. M.; Nason, D. M.; Obach, R. S.; Reinhart, V.; Salomon-Ferrer, R.; Steyn, S. J.; Webb, D.; Yan, J.; Zhang, L. Design and optimization of selective azaindole amide M1 positive allosteric modulators. *Bioorg. Med. Chem. Lett.* **2016**, *26* (2), 650–655. https://doi.org/10.1016/j.bmcl.2015.11.053.

(11) Blass, B. E. Pyrrolo-pyridazine derivatives as muscarinic M1 receptor positive allosteric modulators. *ACS Med. Chem. Lett.* **2021**, *12* (5), 677–678. https://doi.org/10.1021/acsmedchemlett.1c00118.

(12) Kuduk, S. D.; Schlegel, K. A.; Yang, Z. Q. **Merck Sharp & Dohme LLC, assignee.** *Quinoline amide M1 receptor positive allosteric modulators.* U.S. Patent **9,199,939**, December 1, 2015; Merck Sharp & Dohme LLC, Assignee.

(13) Han, C.; Chatterjee, A.; Noetzel, M. J.; Panarese, J. D.; Smith, E.; Chase, P.; Hodder, P.; Niswender, C.; Conn, P. J.; Lindsley, C. W.; Stauffer, S. R. Discovery and SAR of muscarinic receptor subtype 1 (M1) allosteric activators from a molecular libraries high throughput screen. Part 1: 2,5-dibenzyl-2H-pyrazolo[4,3-c]quinolin-3(5H)-ones as positive allosteric modulators. *Bioorg. Med. Chem. Lett.* **2015**, *25* (2), 384–388. https://doi.org/10.1016/j.bmcl.2014.11.011.

(14) Beshore, D. C.; Di Marco, C. N.; Chang, R. K.; Greshock, T. J.; Ma, L.; Wittmann, M.; Seager, M. A.; Koeplinger, K. A.; Thompson, C. D.; Fuerst, J.; Hartman, G. D.; Bilodeau, M. T.; Ray, W. J.; Kuduk, S. D. MK-7622: a first-in-class M1 positive allosteric modulator development candidate. *ACS Med. Chem. Lett.* **2018**, *9* (7), 652–656. https://doi.org/10.1021/acsmedchemlett.8b00095.

(15) Kuduk, S. D.; Chang, R. K.; Greshock, T. J.; Ray, W. J.; Ma, L.; Wittmann, M.; Seager, M. A.; Koeplinger, K. A.; Thompson, C. D.; Hartman, G. D.; Bilodeau, M. T. Identification of amides as carboxylic acid surrogates for quinolizidinone-based M1 positive allosteric modulators. *ACS Med. Chem. Lett.* **2012**, *3* (12), 1070–1074. https://doi.org/10.1021/ml300280g.

(16) Kuduk, S. D.; Di Marco, C. N.; Chang, R. K.; Ray, W. J.; Ma, L.; Wittmann, M.; Seager, M. A.; Koeplinger, K. A.; Thompson, C. D.; Hartman, G. D.; Bilodeau, M. T. Heterocyclic fused pyridone carboxylic acid M1 positive allosteric modulators. *Bioorg. Med. Chem. Lett.* **2010**, *20* (8), 2533–2537. https://doi.org/10.1016/j.bmcl.2010.02.096.

(17) Diaz, P.; Xu, J.; Astruc-Diaz, F.; Pan, H. M.; Brown, D. L.; Naguib, M. Design and synthesis of a novel series of N-alkyl isatin acylhydrazone derivatives that act as selective cannabinoid receptor 2 agonists for the treatment of neuropathic pain. *J. Med. Chem.* **2008**, *51* (16), 4932–4947. https://doi.org/10.1021/jm8002203.

(18) Han, S.; Thoresen, L.; Zhu, X.; Narayanan, S.; Jung, J. K.; Strah-Pleynet, S.; Decaire, M.; Choi, K.; Xiong, Y.; Yue, D.; Semple, G.; Thatte, J.; Solomon, M.; Fu, L.; Whelan, K.; Al-Shamma, H.; Gatlin, J.; Chen, R.; Dang, H.; Pride, C.; Gaidarov, I.; Unett, D. J.; Behan, D. P.; Sadeque, A.; Usmani, K. A.; Chen, C.; Edwards, J.; Morgan, M.; Jones, R. M. Discovery of 1a,2,5,5a-tetrahydro-1H-2,3-diaza-cyclopropa[a]pentalen-4-carboxamides as potent and selective CB2 receptor agonists. *Bioorg. Med. Chem. Lett.* **2015**, *25* (2), 322–326. https://doi.org/10.1016/j.bmcl.2014.11.040.

(19) Sparkes, E.; Cairns, E. A.; Kevin, R. C.; Lai, F.; Grafinger, K. E.; Chen, S.; Deventer, M. H.; Ellison, R.; Boyd, R.; Martin, L. J.; McGregor, I. S.; Gerona, R. R.; Auwärter, V.; Glass, M.; Stove, C.; Banister, S. D. Structure–activity relationships of valine, tert-leucine, and phenylalanine amino acid-derived synthetic cannabinoid receptor agonists related to ADB-BUTINACA, APP-BUTINACA, and ADB-P7AICA. *RSC Med. Chem.* **2022**, *13* (2), 156–174. https://doi.org/10.1039/D1MD00242B.

(20) Manera, C.; Malfitano, A. M.; Parkkari, T.; Lucchesi, V.; Carpi, S.; Fogli, S.; Bertini, S.; Laezza, C.; Ligresti, A.; Saccomanni, G.; Savinainen, J. R.; Ciaglia, E.; Pisanti, S.; Gazzerro, P.; Di Marzo, V.; Nieri, P.; Macchia, M.; Bifulco, M. New quinolone- and 1,8-naphthyridine-3-carboxamides as selective CB2 receptor agonists with anticancer and immunomodulatory activity. *Eur. J. Med. Chem.* **2015**, *97*, 10–18. https://doi.org/10.1016/j.ejmech.2015.04.034.

(21) Lucchesi, V.; Hurst, D. P.; Shore, D. M.; Bertini, S.; Ehrmann, B. M.; Allarà, M.; Lawrence, L.; Ligresti, A.; Minutolo, F.; Saccomanni, G.; Sharir, H.; Macchia, M.; Di Marzo, V.; Abood, M. E.; Reggio, P. H.; Manera, C. CB2-selective cannabinoid receptor ligands: synthesis, pharmacological evaluation, and molecular modeling investigation of 1,8-naphthyridin-2(1H)-one-3-carboxamides. *J. Med. Chem.* **2014**, *57* (21), 8777–8791. https://doi.org/10.1021/jm500807e.

(22) El Bakali, J.; Muccioli, G. G.; Body-Malapel, M.; Djouina, M.; Klupsch, F.; Ghinet, A.; Barczyk, A.; Renault, N.; Chavatte, P.; Desreumaux, P.; Lambert, D. M.; Millet, R. Conformational restriction leading to a selective CB2 cannabinoid receptor agonist orally active against colitis. *ACS Med. Chem. Lett.* **2015**, *6* (2), 198–203. https://doi.org/10.1021/ml500439x.

(23) El Bakali, J.; Muccioli, G. G.; Renault, N.; Pradal, D.; Body-Malapel, M.; Djouina, M.; Hamtiaux, L.; Andrzejak, V.; Desreumaux, P.; Chavatte, P.; Lambert, D. M.; Millet, R. 4-Oxo-1,4-dihydropyridines as selective CB2 cannabinoid receptor ligands: structural insights into the design of a novel inverse agonist series. *J. Med. Chem.* **2010**, *53* (22), 7918–7931. https://doi.org/10.1021/jm100286k.

(24) El Bakali, J.; Gilleron, P.; Body-Malapel, M.; Mansouri, R.; Muccioli, G. G.; Djouina, M.; Barczyk, A.; Klupsch, F.; Andrzejak, V.; Lipka, E.; Furman, C.; Lambert, D. M.; Chavatte, P.; Desreumaux, P.; Millet, R. 4-Oxo-1,4-dihydropyridines as selective CB2 cannabinoid receptor ligands. Part 2: discovery of new agonists endowed with protective effect against experimental colitis. *J. Med. Chem.* **2012**, *55* (20), 8948–8952. https://doi.org/10.1021/jm3008568.

(25) Pasquini, S.; De Rosa, M.; Ligresti, A.; Mugnaini, C.; Brizzi, A.; Caradonna, N. P.; Cascio, M. G.; Bolognini, D.; Pertwee, R. G.; Di Marzo, V.; Corelli, F. Investigations on the 4-quinolone-3-carboxylic acid motif. 6. Synthesis and pharmacological evaluation of 7-substituted quinolone-3-carboxamide derivatives as high affinity ligands for cannabinoid receptors. *Eur. J. Med. Chem.* **2012**, *58*, 30–43. https://doi.org/10.1016/j.ejmech.2012.09.035.

(26) Stern, E.; Muccioli, G. G.; Millet, R.; Goossens, J. F.; Farce, A.; Chavatte, P.; Poupaert, J. H.; Lambert, D. M.; Depreux, P.; Hénichart, J. P. Novel 4-oxo-1,4-dihydroquinoline-3-carboxamide derivatives as new CB2 cannabinoid receptor agonists:

synthesis, pharmacological properties and molecular modeling. *J. Med. Chem.* **2006**, *49* (1), 70–79. https://doi.org/10.1021/jm050467q.

(27) Shi, Y.; Duan, Y. H.; Ji, Y. Y.; Wang, Z. L.; Wu, Y. R.; Gunosewoyo, H.; Xie, X. Y.; Chen, J. Z.; Yang, F.; Li, J.; Tang, J.; Xie, X.; Yu, L. F. Amidoalkylindoles as potent and selective cannabinoid type 2 receptor agonists with *in vivo* efficacy in a mouse model of multiple sclerosis. *J. Med. Chem.* **2017**, *60* (16), 7067–7083. https://doi.org/10.1021/acs.jmedchem.7b00724.

(28) Blaazer, A. R.; Lange, J. H. M.; van der Neut, M. A. W.; Mulder, A.; den Boon, F. S.; Werkman, T. R.; Kruse, C. G.; Wadman, W. J. Novel indole and azaindole (pyrrolopyridine) cannabinoid (CB) receptor agonists: design, synthesis, structure–activity relationships, physicochemical properties and biological activity. *Eur. J. Med. Chem.* **2011**, *46* (10), 5086–5098. https://doi.org/10.1016/j.ejmech.2011.08.021.

(29) Cooper, A. G.; MacDonald, C.; Glass, M.; Hook, S.; Tyndall, J. D. A.; Vernall, A. J. Alkyl indole-based cannabinoid type 2 receptor tools: exploration of linker and fluorophore attachment. *Eur. J. Med. Chem.* **2018**, *145*, 770–789. https://doi.org/10.1016/j.ejmech.2017.11.076.

(30) Spinelli, F.; Capparelli, E.; Abate, C.; Colabufo, N. A.; Contino, M. Perspectives of cannabinoid type 2 receptor (CB2R) ligands in neurodegenerative disorders: structure–affinity relationship (SAfiR) and structure–activity relationship (SAR) studies. *J. Med. Chem.* **2017**, *60* (24), 9913–9931. https://doi.org/10.1021/acs.jmedchem.7b00155.

(31) Bissantz, C.; Grether, U.; Hebeisen, P.; Kimbara, A.; Liu, Q.; Nettekoven, M.; Prunotto, M.; Röver, S.; Rogers-Evans, M.; Schulz-Gasch, T.; Ullmer, C. *Pyridine derivatives as agonists of the CB2 receptor.* U.S. Patent **9,321,727**, **April 26, 2016**.

(32) Nettekoven, M.; Fingerle, J.; Grether, U.; Grüner, S.; Kimbara, A.; Püllmann, B.; Rogers-Evans, M.; Röver, S.; Schuler, F.; Schulz-Gasch, T.; Ullmer, C. Highly potent and selective cannabinoid receptor 2 agonists: initial hit optimization of an adamantyl hit series identified from high-throughput screening. *Bioorg. Med. Chem. Lett.* **2013**, *23* (5), 1177–1181. https://doi.org/10.1016/j.bmcl.2013.01.044.

(33) Lucchesi, V.; Parkkari, T.; Savinainen, J. R.; Malfitano, A. M.; Allarà, M.; Bertini, S.; Castelli, F.; Del Carlo, S.; Laezza, C.; Ligresti, A.; Saccomanni, G.; Bifulco, M.; Di Marzo, V.; Macchia, M.; Manera, C. 1,2-dihydro-2-oxopyridine-3-carboxamides: the C-5 substituent is responsible for functionality switch at CB2 cannabinoid receptor. *Eur. J. Med. Chem.* **2014**, *74*, 524–532. https://doi.org/10.1016/j.ejmech.2013.10.070.

(34) Chicca, A.; Arena, C.; Bertini, S.; Gado, F.; Ciaglia, E.; Abate, M.; Digiacomo, M.; Lapillo, M.; Poli, G.; Bifulco, M.; Macchia, M.; Tuccinardi, T.; Gertsch, J.; Manera, C. Polypharmacological profile of 1,2-dihydro-2-oxo-pyridine-3-carboxamides in the endocannabinoid system. *Eur. J. Med. Chem.* **2018**, *154*, 155–171. https://doi.org/10.1016/j.ejmech.2018.05.019.
